# Engineering Bacterial Secretion Systems for Enhanced Tumor Imaging and Surgical Guidance

**DOI:** 10.1002/adma.202504389

**Published:** 2025-05-21

**Authors:** Dohee Lee, Heung Jin Jeon, Dohyub Jang, Deukhee Lee, Solbi Kim, Minju Han, Sharon Jiyoon Jung, Jung‐Hyun Lee, Jia Choi, Dong Ha Kim, Dong June Ahn, Keri Kim, Sehoon Kim, Hyo‐Jin Lee, SeungBeum Suh

**Affiliations:** ^1^ Bionics Research Center Korea Institute of Science and Technology Seoul 02792 Republic of Korea; ^2^ Department of Chemical and Biological Engineering Korea University Seoul 02841 Republic of Korea; ^3^ Cancer Research Institute Chungnam National University Daejeon 35015 Republic of Korea; ^4^ Chemical and Biological integrative Research Center Korea Institute of Science and Technology Seoul 02792 Republic of Korea; ^5^ Department of Biomicrosystem Technology Korea University Seoul 02841 Republic of Korea; ^6^ Department of Medical Science and Cancer Research Institute Chungnam National University Daejeon 35015 Republic of Korea; ^7^ Technological Convergence Support Center Korea Institute of Science and Technology Seoul 02792 Republic of Korea; ^8^ KU‐KIST Graduate School of Conversing Science and Technology Korea University Seoul 02841 Republic of Korea; ^9^ Department of Chemistry and Nano Science Ewha Womans University Seoul 03760 Republic of Korea; ^10^ Division of Bio‐Medical Science & Technology University of Science & Technology Seoul 02792 Republic of Korea

**Keywords:** fluorescence image‐guided surgery, fluorescent contrast agents, bacteria‐based tumor visualization, streptavidin‐associated *Salmonella* (SAS), surgical guidance

## Abstract

Current imaging techniques suffer from a lack of specificity and resolution, leading to inaccurate tumor imaging and limited applicability of targeted contrast agents, as they require cancer‐specific development. The need for enhanced contrast through improved tumor‐to‐background ratio (TBR) and the toxicity from repeated injections due to fading fluorescent signals further complicate the issue. Additionally, challenges in visualizing the entire 3D tumor with surface‐stained contrast agents highlight the demand for advanced imaging solutions for more precise surgical guidance. A novel approach is proposed utilizing Streptavidin Associated *Salmonella* (SAS) as a contrast agent for image‐guided surgeries. SAS selectively proliferates in cancerous tissues and secretes streptavidin upon induction, enabling the binding of subsequently injected biotin‐conjugated fluorescent dyes. This approach enhances tumor visualization with a TBR of up to 15.3, far surpassing conventional agents (TBR ∼ 2), while enabling prolonged 3‐day imaging, deep tumor penetration, and precise invasive margin delineation with a single contrast agent injection. Furthermore, biosafety evaluations confirmed efficient bacterial clearance, absence of systemic toxicity, and stable physiological responses, supporting its potential for safe clinical translation. This innovative method offers substantial improvements over existing fluorescent contrast agents and holds promise for both diagnostic and therapeutic applications in cancer surgery.

## Graphical abstract

This study presents a novel contrast agent platform, Streptavidin Associated *Salmonella* (SAS), for fluorescence image guided surgery. SAS selectively colonizes tumors and secretes streptavidin, enabling precise delineation of invasive tumor margins with prolonged signal retention. The platform demonstrates robust imaging performance across various tumor types, offering strong potential for clinical translation.

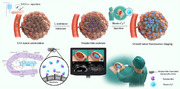

## 1 Introduction

Cancer remains a leading cause of death, accounting for 9.7 million deaths in 2022.^[^ ^1^ ^]^ Surgical resection is a primary treatment strategy for solid tumors; however, accurately distinguishing tumor margins from healthy tissue under white light remains difficult, often resulting in incomplete tumor removal or unnecessary excision of normal tissue.^[^ ^2^, ^3^ ^]^ Current surgeries rely on preoperative imaging (e.g., CT, MRI)^[^ ^4^, ^5^ ^]^ and intraoperative biopsies,^[^ ^3^ ^]^ but these methods are limited in real‐time accuracy. To improve precision, image‐guided surgery using contrast agents that selectively highlight cancerous tissue is being explored. Although modalities such as MRI,^[^ ^4^, ^6^ ^]^ PET,^[^ ^5^, ^7^ ^]^ SPECT,^[^ ^8^ ^]^ and CT^[^ ^4^, ^5^, ^7^, ^8^ ^]^ are used, their intraoperative application is hindered by bulky equipment, radiation exposure, and limited resolution at tumor margins.^[^ ^6^, ^9^, ^10^, ^11^, ^12^ ^]^ Fluorescence‐guided surgery (FGS) using optical contrast agents offers real‐time, high‐resolution imaging as a promising alternative.^[^ ^13^ ^]^ However, current agents often require repeateddosing and lack specificity, particularly in imaging hypoxic or necrotic tumor regions located deep within the tissue.^[^ ^5^, ^10^, ^13^, ^14^, ^15^ ^]^ Moreover, most agents are designed for cancer type–specific targets, limiting their generalizability. There is a critical need for tumor‐agnostic contrast agents that can achieve deep, stable, and three‐dimensional tumor visualization to guide complete and precise tumor resection.

**Figure 1:**
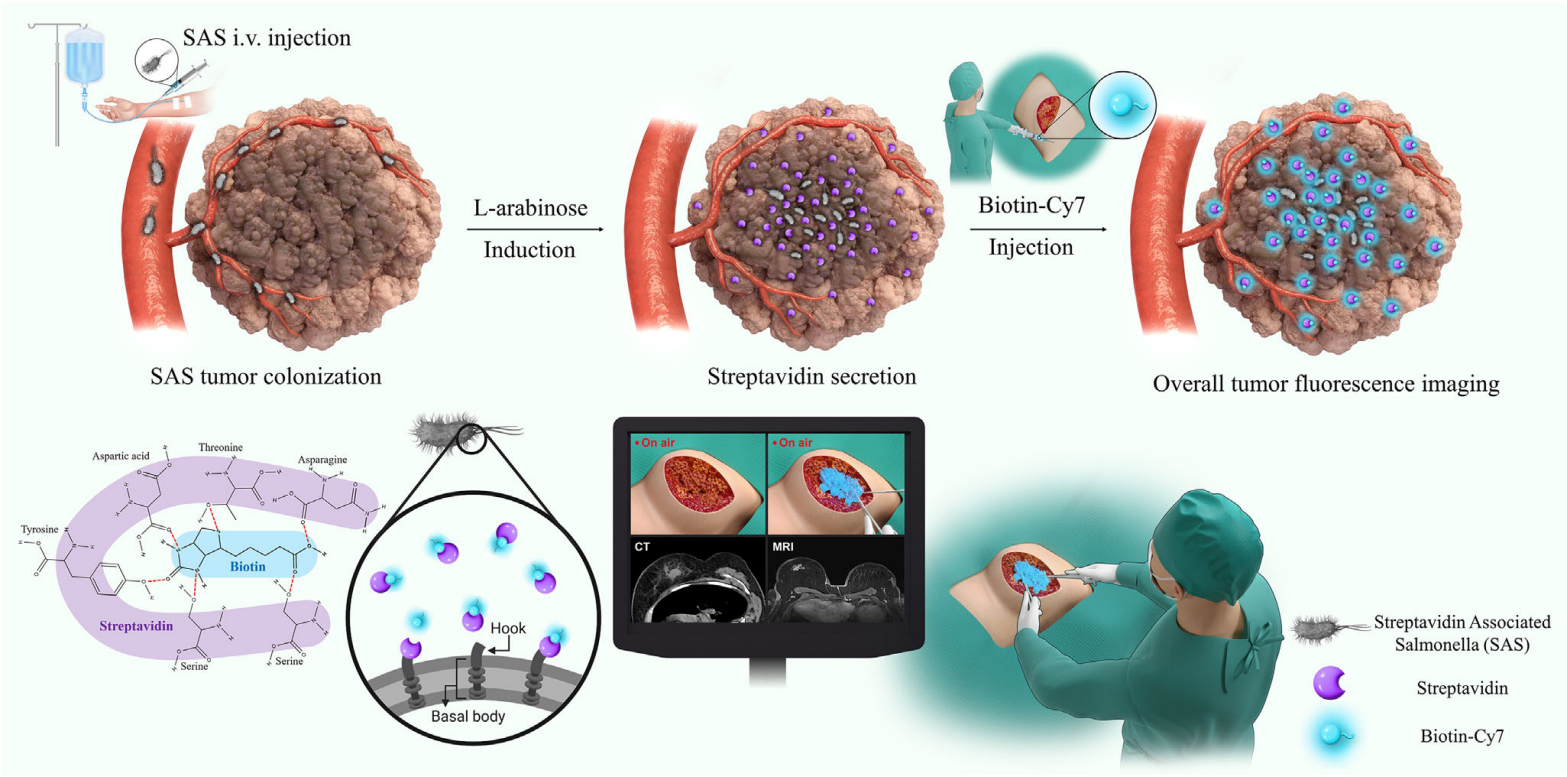
Schematic for fluorescence‐guided surgery system based on Streptavidin‐Associated *Salmonella*.

In this study, we employed bacteria as a fluorescent contrast agent to aid in tumor resection surgeries (**Figure** **1**). Bacteria are known to selectively accumulate in cancer tissues and penetrate deep into the tumor core. Since the early 2000s, various bacterial strains, including *Salmonella typhimurium* and *Escherichia coli*, have been explored for cancer treatment.^[^ ^16^, ^17^, ^18^, ^19^, ^20^ ^]^ Advances in genetic recombination and plasmid synthesis have enabled the stable construction of recombinant bacteria that, once injected, interact with the host immune system, infiltrating and proliferating in the hypoxic regions of tumor.^[^ ^21^, ^22^, ^23^, ^24^ ^]^ These hypoxic regions, including necrotic areas, shield anaerobic bacteria from immune cells, making them effective in targeting cancerous tissues irrespective of cancer type.^[^ ^17^, ^25^ ^]^ Unlike antibody‐based streptavidin‐biotin tumor pre‐targeting imaging methods, which rely on targeting specific antigens expressed on viable tumor cells,^[^ ^14^ ^]^ our approach leverages the ability of streptavidin‐associated *Salmonella* (SAS) to colonize the tumor microenvironment, including hypoxic and necrotic regions, overcoming inter‐tumoral heterogeneity and ensuring broader and more consistent imaging (International Journal of Pharmaceutics 605 (2021)). We developed a SAS strain by engineering *Salmonella typhimurium*, a bacterium known for its cancer‐targeting ability. SAS selectively proliferates in cancerous tissue and using an induction system, secretes streptavidin, which binds to subsequently injected biotin‐conjugated fluorescent dye, effectively highlighting the cancerous tissue. This bacterial system enables deep tumor penetration and streptavidin secretion, which diffuses outward due to high interstitial tumor pressure, allowing for uniform streptavidin distribution throughout the tumor. This streptavidin‐biotin conjugation system produced a significantly higher fluorescent signal intensity in cancerous tissue compared to adjacent normal tissue (TBR ∼ 15.3), allowing for prolonged imaging.^[^ ^13^, ^26^, ^27^, ^28^ ^]^ The three‐dimensional penetration and proliferation of the bacteria within the tumor extended streptavidin distribution deep into the cancer, enabling comprehensive imaging of the entire tumor mass.^[^ ^21^, ^22^, ^23^, ^24^ ^]^ By leveraging bacterial penetration into the tumor core rather than solely targeting surface antigens, our approach enhances tumor imaging beyond the limitations of current strategies. The bacteria's natural propensity to target and proliferate in the hypoxic regions of the tumor microenvironment underscores its broad applicability in imaging diverse cancer types, surpassing the limitations of single‐phenotype targeting strategies.^[^ ^17^, ^25^ ^]^ The effectiveness of the SAS‐based imaging strategy was evaluated in tumor bearing mouse models, confirming its specificity in delineating tumor during surgical resection. Additional biosafety evaluations confirmed effective bacterial clearance, absence of systemic toxicity, and stable physiological responses, supporting its potential for clinical translation. To our knowledge, this study represents the first attempt to develop bacterial contrast agents for image‐guided surgery, offering an innovative modality with distinct advantages over conventional contrast agents.

## 2 Results

### 2.1 Engineering of flgM‐Streptavidin Fusion Genes for Stable and Efficient Secretion via the Flagella Type III Secretion System (T3SS)

To develop a payload delivery system for tumor‐targeting bacteria, we constructed a plasmid that enables streptavidin secretion via the flagella T3SS.^[^ ^29^, ^30^ ^]^ While conventional T3SS directly injects proteins into host cells, the flagella‐associated T3SS facilitates external protein secretion through flagellar assembly, making it an ideal choice for delivering functional proteins.^[^ ^30^ ^]^ This system has also been effectively applied for the selective purification of recombinant neuroactive peptides.^[^ ^29^ ^]^

**Figure 2:**
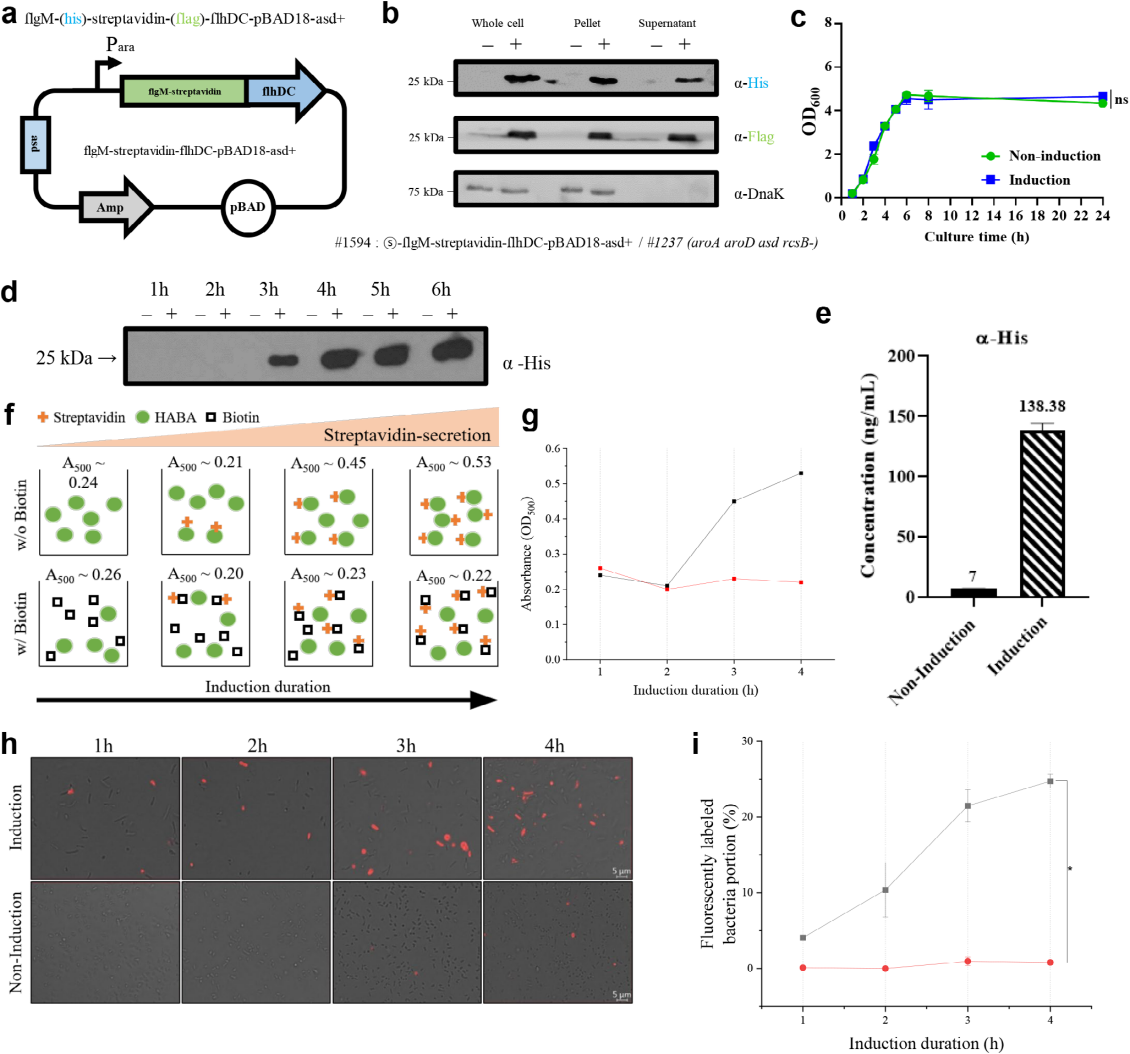
Engineering of *Salmonella* expressing flgM‐streptavidin fusion proteins. a) Plasmid map of flgM‐streptavidin for the expression and secretion of recombinant streptavidin, tagged with flgM at the N‐terminus and 6x His. b) *Salmonella* secreting FlgM‐Streptavidin was incubated in LB at 37 °C for 1 h, and secretion was induced by 0.04% L‐arabinose. After 6 h of further incubation, equal amounts of whole cells, pellet, and supernatant were obtained, and expression was analyzed by western blot using an anti‐His antibody and anti‐Flag antibody. Anti‐DnaK antibody was used as a cell lysis control. c) Growth curve (OD_600_) measurement. (*n* = 3) Data are presented as the mean ± SEM. d) Western blot analysis using an anti‐His antibody to assess secretion at indicated time points after L‐arabinose induction in a strain designed to secrete streptavidin. e) Quantification of FlgM‐Streptavidin using anti‐His ELISA in samples obtained at the end of L‐arabinose induction. f) Schematic for streptavidin secretion verification using a HABA assay. g) Absorbance at 500 nm (OD_500_) varying through streptavidin secretion when bound to HABA. (red: w/ biotin‐Cy7 & black: w/o biotin‐Cy7). h) Verification of streptavidin functionalization on the bacterial surface through biotin‐Cy7 dye staining (scale bar = 5 µm). i) Fluorescently labeled bacteria showing the proportion of bacteria secreting streptavidin. (red: w/o L‐arabinose & black: w/ L‐arabinose) Statistical analyses were performed using paired t‐tests. (*p < 0.05).

For efficient secretion, streptavidin was translationally fused to the C‐terminal of FlgM, a protein naturally secreted via the flagella T3SS (Figure S1a, Supporting Information).^[^ ^29^ ^]^ The flgM‐streptavidin gene was incorporated into a pBad‐based inducible expression system (**Figure** **2**a), enabling controlled expression upon L‐arabinose induction.^[^ ^31^, ^32^ ^]^ To further enhance secretion efficiency, the flhDC gene, which a master regulator involved in flagellar biosynthesis, was inserted downstream of the flgM‐streptavidin gene.^[^ ^29^, ^30^ ^]^

To maintain plasmid stability and ensure survival in vivo, we implemented a balanced‐lethal system using an asd gene complementation strategy.^[^ ^33^ ^]^ The host bacterial strain (#177) lacks the asd gene, rendering it dependent on plasmids carrying the functional asd gene for survival (Table S1, Supporting Information). Because diaminopimelic acid (DAP) is absent in mammalian tissues, this system ensures stable plasmid retention without external antibiotic selection (Figure 2a; Table S1, Supporting Information^[^ ^29^ ^]^). This genetic engineering strategy effectively prevents plasmid loss, ensuring long‐term genetic stability in the host bacteria while enabling the controlled and sustained secretion of streptavidin for its functional application as a tumor‐targeting bacterial contrast agent.

### 2.2 Confirmation of FlgM‐Streptavidin Secretion

#### 2.2.1 Confirmation of FlgM‐Streptavidin Secreting *Salmonella*

We completed the engineering of flgM‐streptavidin expressing *Salmonella* strain #1237 (Table S1, Supporting Information) by transforming the aforementioned plasmid into it (Figure 2a). This resulted in the engineered *Salmonella* strain #1594. To validate the secretion of FlgM‐Streptavidin, we induced the strain with L‐arabinose for 6 h, after which the samples were divided into three parts: whole cells, pellet, and supernatant. We confirmed the expression and secretion of FlgM‐Streptavidin in the three parts using anti‐His (internal tag), anti‐FLAG (C‐terminal tag) and anti‐DnaK antibodies (Figure 2b; Figure S1, Supporting Information). Clear bands were observed with both anti‐His and anti‐FLAG antibodies across all fractions, indicating successful expression and secretion of FlgM‐Streptavidin. However, the chaperone protein DnaK was detected in the whole cells and pellets but absent in the supernatant. This suggests that the engineered *Salmonella* strain actively secretes FlgM‐Streptavidin into the medium via the Flagellar T3SS, rather than passively releasing it upon cell death, thus confirming its active secretion capability.^[^ ^29^, ^30^ ^]^

#### 2.2.2 Streptavidin Secretion Mechanism and Quantification, and Verification of Biotin Binding

To validate the secretion mechanism and quantify streptavidin secretion, we monitored the growth of the strain containing the flgM‐streptavidin plasmid by measuring the optical density at 600 nm (OD_600_) at different time points, both with and without induction using 0.2% L‐arabinose (final concentration) at 1 h (Figure 2c). At 6 h post‐induction, the OD_600_ readings for both the induced and uninduced samples were comparable, with values of 0.43 and 0.40, respectively, after being diluted 10‐fold. This indicates that L‐arabinose induction did not have a significant impact on cell growth. Upon an anti‐His ELISA assay, secreted streptavidin was first detected 3 h post‐induction, with concentrations progressively increasing over time (Figure 2d). At 6 h post‐induction, the induced sample exhibited about 20‐fold higher secretion level (138.38 ng/mL) compared to the uninduced sample (7 ng/mL) (Figure 2e).

We then evaluated the impact of FlgM‐Streptavidin secretion on bacterial motility through a motility assay (Figure S2a, Supporting Information). Strain 1, containing an empty plasmid, was compared to strain 2 (#1594 strain), containing the flgM‐streptavidin plasmid. In the absence of induction, both strains exhibited active motility. However, upon induction with L‐arabinose, strain 2 showed reduced motility, indicating that flgM‐mediated secretion of streptavidin affected the flagellar proteins (fliC or fljB), as confirmed by SEM imaging (Figure S2b, Supporting Information).^[^ ^29^ ^]^

To investigate the correlation between the binding efficiency of secreted streptavidin with biotinylated fluorescent dye (biotin‐Cy7) and the bacteria induction time, we employed the HABA (4′‐hydroxyazobenzene‐2‐carboxylic acid) assay. The HABA assay is traditionally used to verify biotinylation, where the binding of HABA to streptavidin results in an increase in absorbance at 500 nm. In the presence of biotin, however, the stronger biotin‐streptavidin interaction displaces HABA, leading to a decrease in absorbance. In this experiment, we compared conditions with and without excess biotin to verify the binding between biotin and streptavidin secreted by bacteria, as indicated by absorbance changes. The results showed that streptavidin secretion and binding increased from 2 h post‐induction, reaching saturation at 4 h, which informed our in vivo imaging protocol. Based on this finding, biotin‐conjugated fluorescent dyes were injected 2 hours after induction, and imaging was performed at 4 hours post‐induction to ensure maximal streptavidin availability for effective tumor imaging (Figure 2f, g).

Streptavidin is secreted extracellularly through the basal body and hook of the flagella on the bacterial surface (enlarged in Figure 1). As a result, streptavidin not only gets secreted outside the cell but also functionalizes the bacterial surface. To quantify the binding between surface‐functionalized streptavidin on the bacteria and biotinylated fluorescent dye, we measured the portion of bacteria exhibiting fluorescent signals from conjugated biotin‐Cy7. As the induction time increased, the proportion of fluorescently labeled bacteria increased, and after 4 h, more than 25% of the bacterial surface exhibited bound fluorescent signals. This confirms that SAS can continuously secrete and activate streptavidin, highlighting its potential as an effective contrast agent.

### 2.3 Validation of Enhanced Intra‐Tumoral Fluorescence Signal Retention via SAS

**Figure 3:**
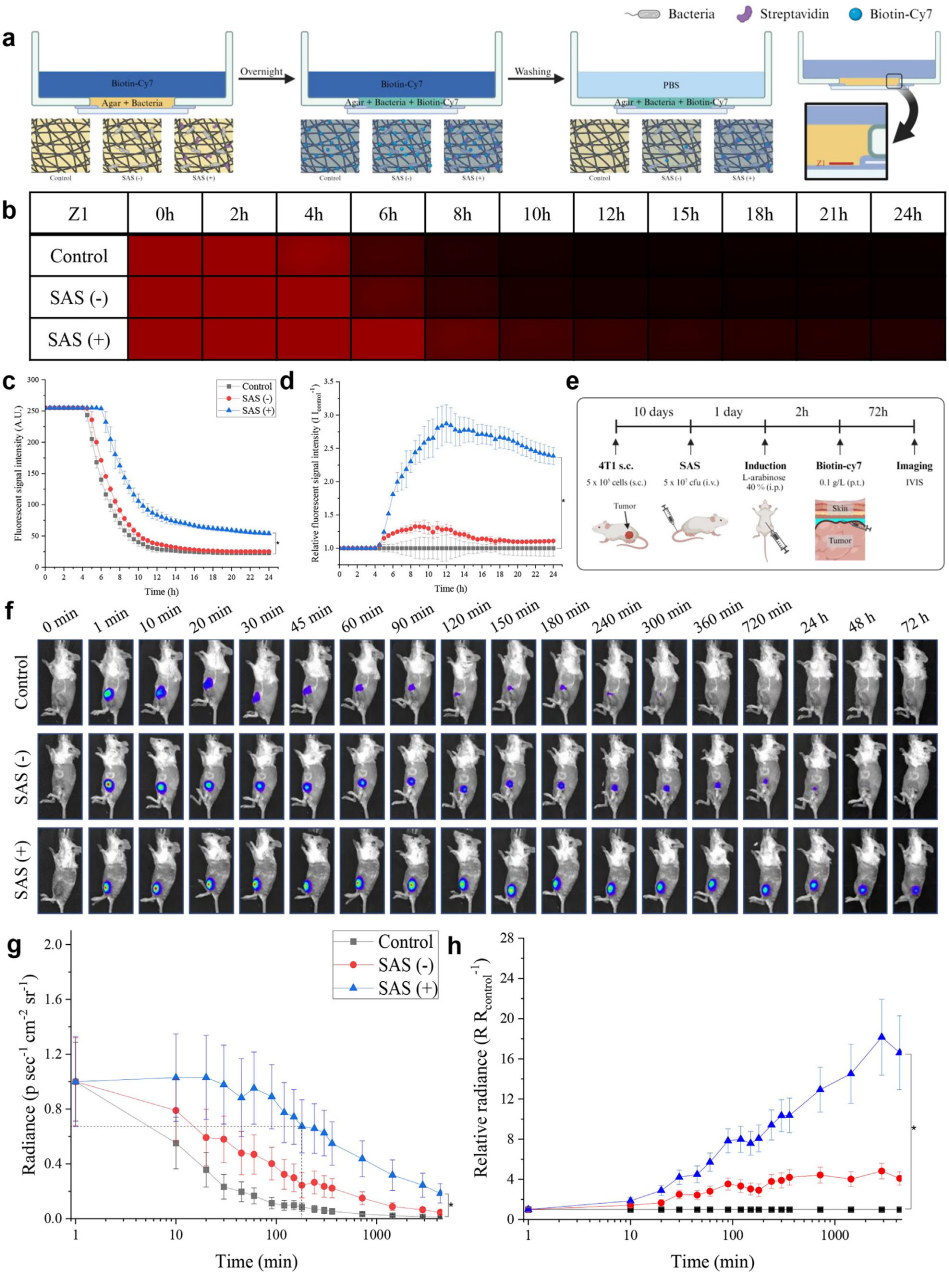
Validation of enhanced intra‐tumoral fluorescent signal retention via SAS. a) Schematic diagram of the in vitro experiment using agar. b) Time‐dependent fluorescence images at the Z1 position from (a). c) Fluorescent signal intensity profile (0‐255) from (b). (*n* = 3) (d) Fluorescent signal intensity profile from (b) normalized to the control group. (*n* = 3). e) Schematic diagram of the in vivo experiment. f) IVIS images of mice over time (filter set: λ_*exc*_ = 745 nm, λ_*emi*_ = 800 nm). g) Time‐dependent fluorescent signal intensity in the tumor, normalized to the initial radiance value, with a dotted line indicating 3 h for reference. (*n* = 3) h) Relative fluorescent signal from (e), normalized to the control group. (*n* = 3) Statistical analysis was performed using paired *t*‐tests, comparing SAS (+) and control groups. (*p < 0.05).

In fluorescence image‐guided surgery, where fluorescent contrast agents are used to locate tumors, blood flow and continuous drainage often dilute the contrast agents, reducing the signal intensity and requiring repeated dye injections. The proposed SAS approach enhances retention in cancerous tissues by chemically binding secreted streptavidin to biotinylated fluorescent dyes, thereby anchoring the signals within the tumor for an extended time. To verify the sustained presence of biotin‐Cy7 bound to streptavidin compared to free biotin‐Cy7 under continuous drainage conditions, we conducted tumor microenvironment simulation experiments using agar (**Figure** **3**a). Agar, mimicking the tumor environment,^[^ ^34^ ^]^ allowed us to monitor fluorescent signal reduction in the absence (control) and presence of either induced (SAS (+)) or uninduced (SAS (−)) bacteria (Figure 3b–d). The control and SAS (−) groups showed a rapid fluorescence decline starting at 4 h, whereas this decline point was delayed to 6 h in the SAS (+) group. Notably, the SAS (+) group displayed a slower decline rate compared to the others, ascribed to the secretion of streptavidin upon induction, which slows down the washout of biotin‐Cy7 due to the enlarged molecular size resulting from its binding to secreted streptavidin. At 24 h, when fluorescent signal intensity reached equilibrium, both the control and SAS (−) groups exhibited similarly low levels of residual signals, while the SAS (+) group maintained a fluorescent intensity more than 2.5 times higher than that of the control (Figure 3d). This signal difference at equilibrium is attributed to the binding of biotin‐Cy7 to bacterial surface‐expressed streptavidin, which persisted longer within the agar matrix along with bacteria. These findings confirm that bacteria‐based contrast agents provide an enhanced and prolonged fluorescent signal intensity relative to traditional molecular contrast agents. Additional experiments also showed that the SAS (+) group exhibited slower drainage rates across all agar depths, suggesting that streptavidin secretion by bacteria in deeper tumor regions effectively delays lymphatic drainage of imaging agents, thereby enhancing sustained contrast (Figure S3, Supporting Information).

We further investigated the enhancement in imaging performance in vivo by monitoring temporal changes in fluorescent signal intensity in 4T1 tumor‐bearing Balb/c mice, following sequential procedures composed of intravenous injection of SAS, intraperitoneal injection of L‐arabinose, and peritumoral injection of biotin‐Cy7 (Figure 3e). Consistent with the in vitro results, the SAS (+) group maintained significantly higher fluorescent signal intensity within the tumor, compared to the control and SAS (−) groups. Sustained tumor signals were observed throughout the experimental period of up to 72 h (Figure 3f, g), exhibiting the substantial advantage of our SAS approach in achieving persistent contrasts for tumor imaging. The relative radiance of the SAS (+) group, normalized by the control, increased over time, reaching up to nearly 20 times higher fluorescent signal intensity (Figure 3h). This demonstrates that tumor imaging using SAS provides significantly enhanced fluorescent contrast effects over time, surpassing conventional contrast agents. When approximating the decay of tumor signals with Equation (1), *A*_*i*_ quantifies the contribution from distinct states of Cy7 (i.e., free biotin‐Cy7, secreted streptavidin‐bound biotin‐Cy7, bacteria‐bound biotin‐Cy7, and tissue‐adsorbed biotin‐Cy7 etc.), while τ_*i*_ represents the time constant for the clearance of each state from the tumor (Table S2, Supporting Information).In the control group, over 60% of the tumor signal dissipated from the outermost tumor layer within 10 min, with only about 12% remaining after 6 h. In contrast, more than 60% of the signal in the SAS (+) group was retained for over 542 min (∼9 h), with over 53% located in the deepest tumor regions, persisting for up to 16,727 min (∼ 11 days). Considering that the average surgical time for breast cancer is within 3–4 h, bacteria‐based tumor monitoring in the SAS (+) group demonstrated that more than 65% of cases exhibited late decay (dotted line in Figure 3g), providing sufficient fluorescent contrast for the entire duration of surgery with a single injection of the fluorescent contrast agent.^[^ ^35^ ^]^

(1) $$y\left(t\right)=\sum_{i=1}^{n}\left(A_{i}e^{\left(-\frac{t}{\tau_{i}}\right)}\right)+y_{0}$$

### 2.4 Tumor‐Selective Imaging and Enhanced Contrast Efficiency of SAS

**Figure 4:**
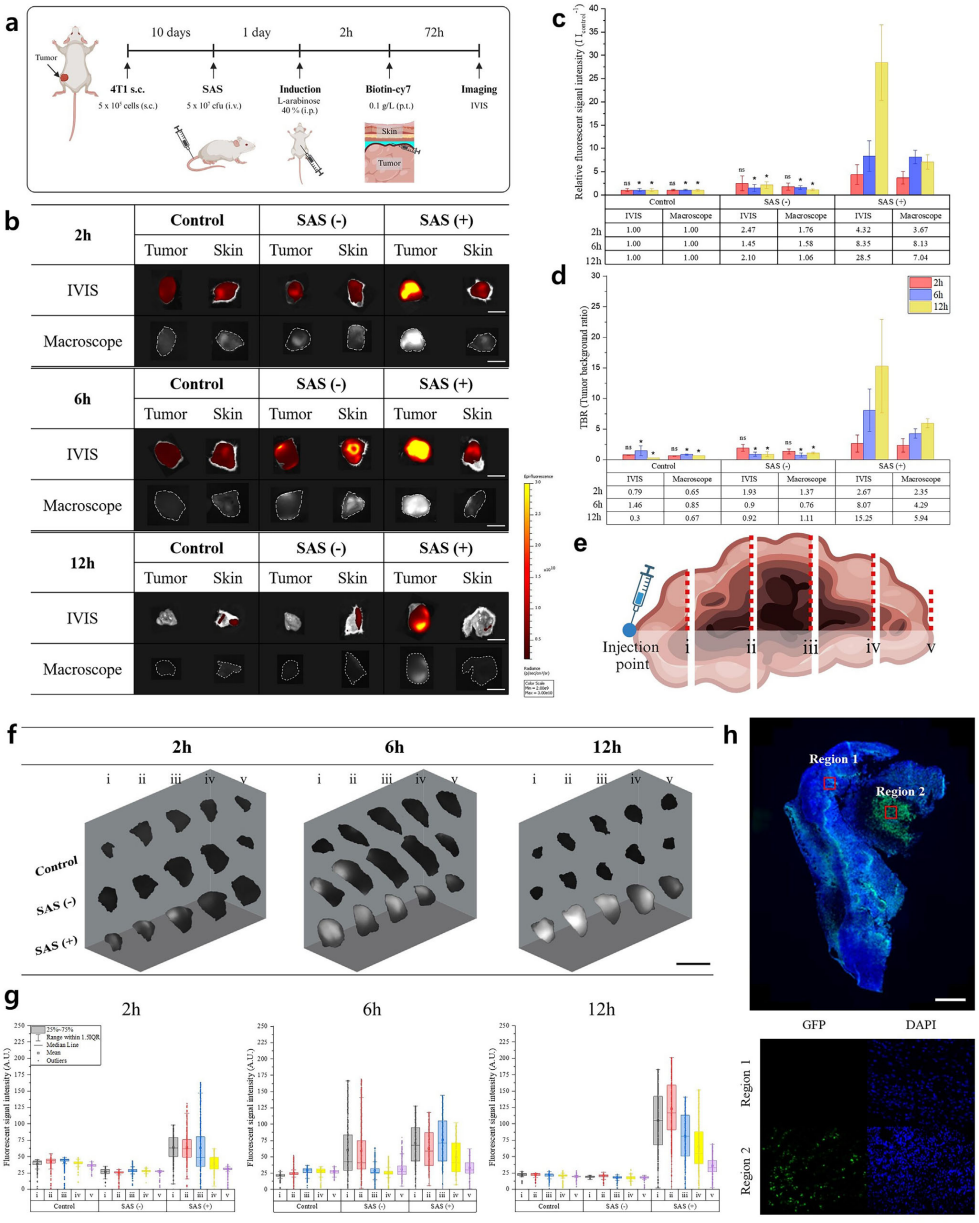
Tumor visualization and 3D contrast enhancement via SAS penetration. a) Schematic diagram of the in vivo experiment. b) Fluorescence images of tumors and adjacent skin captured using IVIS and a macroscope at 2, 6, and 12 h post‐incubation (Scale bar = 1 cm). c) TBR, with statistical comparisons between the SAS(+) group and each of the other individually. (*n* = 3) d) Relative fluorescent signal intensity of the tumor, normalized to each respective control group, with statistical analysis results included. (*n* = 3) e) Schematic diagram of the three‐dimensional visualization experiment. f) Macroscope fluorescence images of the tumor after dissection into five equal parts (Scale bar = 1 cm). g) Average fluorescent signal intensity (0‐255) across the dissected surfaces. h) Bacterial selective colonization in the hypoxic region of a 4T1 tumor, with region 1 indicating the active tumor area and region 2 indicating the hypoxic region (blue: DAPI staining; green: SAS with FITC plasmid, scale bar = 1mm). Statistical analyses were performed using paired t‐tests, comparing control and SAS (−) to SAS (+) groups. (*p < 0.05).

Accurate differentiation of tumor from surrounding normal tissues is critical for the effectiveness of contrast agents used in tumor resection surgery, as the success of the procedure depends on the clear contrast between the fluorescent signals emitted by the tumor and those from surrounding tissues. Typically, the efficacy of these contrast agents is quantified using the tumor‐to‐background ratio (TBR), which normalizes the fluorescent signal intensity from the tumor relative to that of the surrounding tissue. Under the in vivo experimental conditions, animal studies were conducted to measure the fluorescent signal intensity in the tumor, surrounding skin, and other organs (liver, spleen, and kidney) at specified time points (2, 6, and 12 h) (**Figure** **4**a). These measurements were taken using both an in vivo imaging system (IVIS) and a custom‐built fluorescent macroscope designed for potential intraoperative application (Figure S4, Supporting Information). The TBR was calculated by normalizing the fluorescent signal intensity of the tumor to that of the surrounding skin tissue.

At all time points, the SAS (+) group exhibited significantly higher tumor signals compared to the control and SAS (−) groups (Figure 4b). Quantitative analysis, normalized to tumor signals in the control group, revealed that the SAS (+) group exhibited fluorescent signal intensities 4.3‐fold, 8.4‐fold, and 28.5‐fold higher at 2, 6, and 12 h post‐injection, respectively (Figure 4c; Figure S5, Supporting Information). This trend is further clarified by examining the TBR over time. In the control and SAS (−) groups, TBR values remained close to or below 1, indicating limited tumor contrast due to the weak tumor signals comparable to autofluorescence from the surrounding skin. In contrast, the SAS (+) group maintained high fluorescent signal intensity, resulting in a progressively increasing TBR over time (Figure 4d). At 12 h post‐injection, the TBR reached 15.25, a significant improvement compared to conventional systems, where a peak TBR of 5.2 or higher is considered excellent.^[^ ^28^, ^36^ ^]^ Given that the average duration of breast cancer surgery is 3–4 h and a TBR of 2 or higher is typically sufficient for effective contrast, maintaining a TBR above 2 for 12 h with a single injection of the contrast agent eliminates the need for repeated administrations, potentially reducing toxicity.^[^ ^28^, ^37^, ^38^, ^39^, ^40^, ^41^ ^]^

### 2.5 Enhanced 3D Contrast Efficacy via Intratumoral Penetration of SAS

During tumor resection surgery, distinguishing cancerous tissues is challenging, as they are gradually excised starting from the skin tissue incision.^[^ ^42^ ^]^ Current contrast agents, which primarily stain the tumor periphery, are also limited in accurately delineating the three‐dimensional surface‐to‐core structure of the entire tumor, due to the restricted diffusion within the tumor microenvironment. This makes it difficult to obtain a precise understanding of the tumor's full extent during surgery.^[^ ^2^, ^3^ ^]^ To address this challenge, we facilitated bacterial infiltration and colonization into the deeper regions of tumor to visualize its three‐dimensional structure, including the core. To simulate the gradual excision of tumor during surgery, we divided the tumor depthwise into five sections, as shown in Figure 4e, and performed imaging and fluorescent signal intensity analysis of each slice using a macroscope.

Fluorescent signal intensity was significantly higher in all cross‐sections of the SAS (+) group compared to the other groups, suggesting that only the SAS (+) group is capable of fluorescently delineating the entire three‐dimensional structure of the tumor (Figure 4f, g). This trend became more pronounced over time, with fluorescence retention observed even in the deepest tumor regions at 6 and 12 h. In contrast, the control and SAS (−) groups showed overall low fluorescent signals in the deeper tumor regions, making it difficult to assess the full extent of the tumor. This suggests that higher TBR observed in the SAS (+) group, as measured by the surrounding tissue contrast, is likely to enhance the effectiveness of tumor visualization during the surgical process, particularly as incisions are made deeper into the tumor. The high fluorescent signal intensity concentrated in the tumor core indicates a fluorescence enhancement effect, likely driven by bacterial selective colonization, as shown in the bacteria experiments (Figure S6a, Supporting Information). This effect is particularly pronounced in the deeper, hypoxic regions of the tumor (Figure 4h). In the SAS (+) group, the fluorescent signal observed at 2 h was concentrated in the tumor core, with a tendency to diffuse throughout the entire tumor over time (Figure 4f). This pattern indicates that streptavidin secreted in the hypoxic core initially binds to biotin‐Cy7, maintaining its localization, and subsequently migrates toward the tumor periphery due to elevated interstitial pressure from the center.^[^ ^6^, ^12^, ^43^ ^]^ Interestingly, the dense, hypoxic tumor microenvironment, which typically impedes the delivery of conventional contrast agents, conversely proves advantageous for bacteria‐based agents, facilitating effective visualization of the entire three‐dimensional structure of the tumor.

### 2.6 SAS Mediated Fluorescence Image‐Guided Tumor Resection Surgery

**Figure 5:**
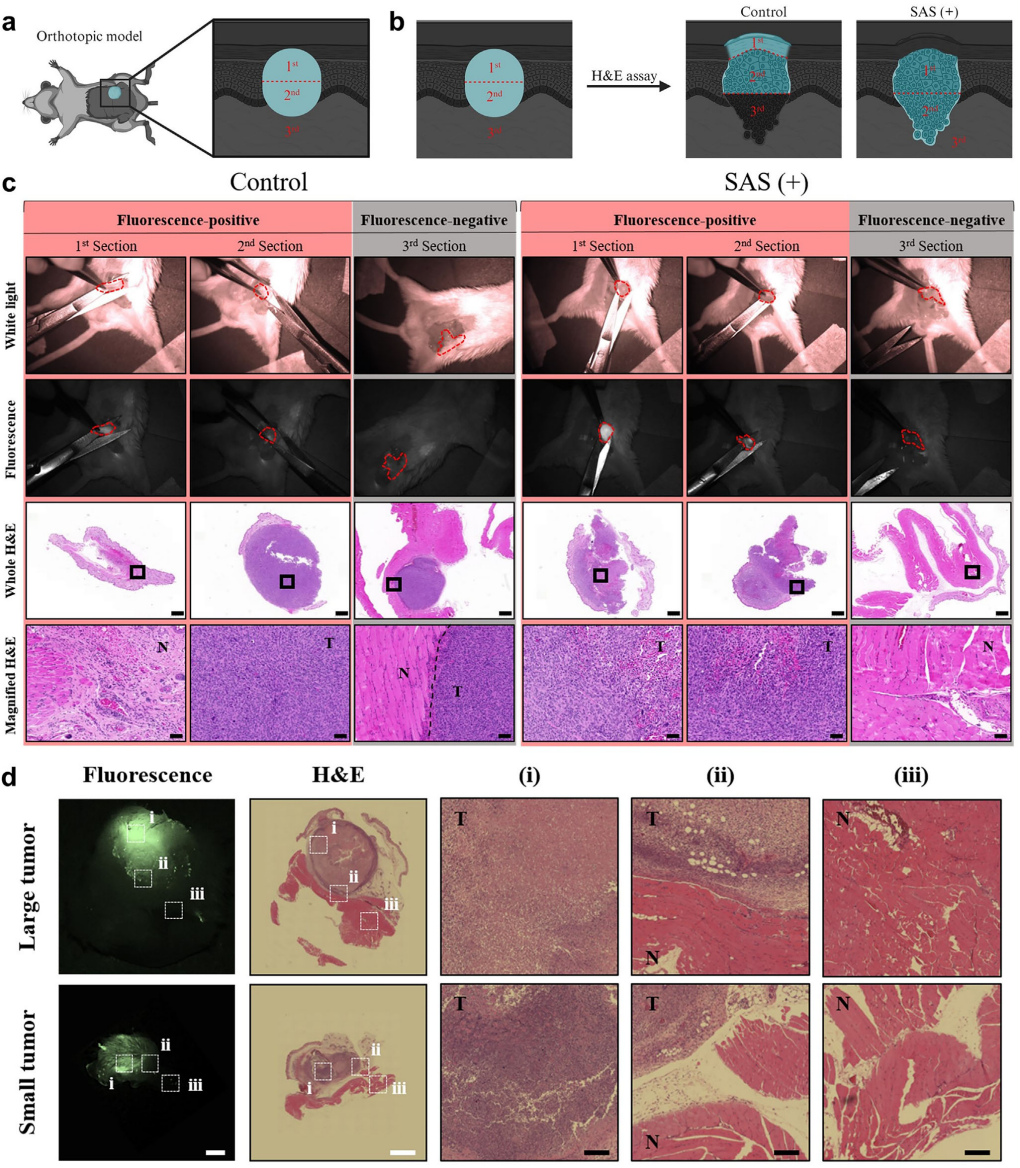
Analysis of surgical tissues to confirm the accuracy of FGS in the orthotopic 4T1 tumor model. Fluorescent areas were excised in two steps during surgery, while a non‐fluorescent surrounding area was excised once for analysis. a) Schematic representation of FGS using the orthotopic mouse model. b) Schematic illustration of the actual excised tissue based on the H&E image analysis from (c). c) Bright field and fluorescence filter images captured during surgery, with red dotted lines indicating excised tissues. H&E images of the excised tissues (scale bar = 500 µm) and magnified images of the boxed areas (scale bar = 50 µm) (T: Tumor, N: Normal tissue). d) Fluorescence images and H&E images in different tumor sizes (white scale bar = 2 mm, black scale bar = 200 µm) (T: Tumor, N: Normal tissue).

The efficacy of tumor resection under FGS with SAS was assessed in an orthotopic 4T1 breast cancer mouse model which closely mimics the physiological tumor environment, including its invasive nature. During tumor excision, tissues displaying fluorescence were resected continuously until no further fluorescence was detected (**Figure** **5**a; Movie S1, Supporting Information). Surrounding non‐fluorescent areas were also excised to verify the correlation between the fluorescent signal and the presence of remaining cancerous tissue. Histological analysis revealed that, in the control group imaged with a fluorescent dye, both cancerous and normal tissues were present in fluorescent‐positive regions. Additionally, fluorescent dyes that were unable to penetrate the tumor due to the elevated pressure in the tumor core remained outside the tumor, leading to fluorescent signals being observed in normal tissues, similar to what was seen in the first section of the control case. However, in the group imaged with SAS (+), all tissues exhibiting fluorescence were identified as tumor (1^st^ and 2^nd^ sections in Figure 5b; Figure S7, Supporting Information). Following the removal of fluorescence‐positive tissues presumed to be tumors, the remaining non‐fluorescent tissues (3^rd^ section) were examined. In the control group, residual cancerous tissues were still detected alongside normal tissues, whereas in the SAS (+) group, only normal tissues were identified, indicating complete tumor resection (Figure 5c).

To validate the accuracy of tumor margin delineation, particularly in capturing the invasive nature of malignant tumor margins, we compared the tumor‐positive margin predicted solely by fluorescent signals with the true tumor boundary confirmed by H&E staining. Since minimizing the positive margin is critical for tumor surgery, ensuring accurate localization of the tumor‐invasive boundary is essential. Plus, tumors are known to be highly heterogeneous, with varying levels of hypoxia, necrosis, and vascularization, which could influence the performance of contrast agents. To evaluate whether SAS maintains its imaging performance in tumors with different structural characteristics, we assessed its performance in orthotopic tumor models of varying sizes. Larger tumors generally exhibit more pronounced hypoxic regions and increased structural heterogeneity, which can impact contrast agent distribution. Despite these differences, the fluorescence‐positive tumor margins in both small and large tumors showed strong alignment with histological H&E staining, confirming reliable tumor boundary delineation across different tumor sizes (Figure 5d). These findings suggest that while tumor heterogeneity is multifaceted, SAS‐based fluorescence imaging effectively delineates tumor‐invasive boundaries with varying degrees of size‐related structural differences. Conventional fluorescent dyes failed to penetrate the tumor core within 6 hours, leading to signal dispersion in surrounding tissues and inaccurate margins. In contrast, the SAS system maintained fluorescence retention, effectively guiding tumor resection and minimizing positive margins. Additionally, SAS selectively colonizes the tumor microenvironment (TME), which differs from general inflammatory sites due to its hypoxic and immunosuppressive characteristics. Preoperative protocols help mitigate inflammation‐related confounding factors, further reducing the risk of non‐specific imaging,^[^ ^44^ ^]^ and even if bacteria colonize inflamed tissues, the localized administration of biotinylated fluorescent contrast agents ensures tumor‐specific imaging, minimizing false‐positive signals. These results confirm the potential of SAS as a precise fluorescence‐guided imaging tool for tumor resection.

### 2.7 Long‐Term Biosafety Evaluation

To evaluate the biosafety of the SAS system, we conducted a comprehensive analysis of its systemic effects, including overall toxicity. Before *Salmonella* injection for biosafety experiment, tumor sizes were similar across all groups (150–200 mm^3^). After intravenous administration, streptavidin secretion was induced via intraperitoneal L‐arabinose injection starting on day 3 (Figure 5a). Body weight was monitored periodically, showing initial weight loss until day 2, but body weights fully recovered within five days and remained stable throughout treatment, indicating the safety of live *Salmonella* administration (Figure 5b). On day 15, when the PBS group tumors reached 1500 mm^3^, body weights in the *Salmonella*‐treated groups remained stable, further confirming the tolerability of the treatment.

Blood samples collected on day 15 were analyzed for inflammation markers, showing TNF‐α and IL‐6 levels within the normal range (Figure 5c,d). These results indicate that bacterial colonization remained localized within the tumor and did not trigger long‐term systemic inflammation. General biochemical markers (CREA, BUN, ALT, AST) also remained within normal limits (Figure 5e–h). Histopathological examination via H&E staining revealed no observable lesions (Figure 5i), suggesting *Salmonella* did not induce acute toxicity and selectively targeted tumor tissues without affecting normal tissues. Spleen size analysis showed transient splenomegaly, which normalized over time (Figure S8a, Supporting Information). These findings highlight *Salmonella*'s potential as a safe and effective tumor‐targeting system with minimal toxicity.

To further evaluate the biosafety of the SAS system, we analyzed its pharmacokinetics, specifically the in vivo stability and clearance of the streptavidin‐biotin complex, by quantifying the concentration of continuously secreted streptavidin in the blood at different time points using ELISA, following bacterial induction (Figure S8b, Supporting Information). Streptavidin was detected at low concentrations in the picomolar range on day 1, increasing to an average of 13 pM by day 3 before becoming undetectable by day 7. Blood samples were collected at 1, 3, 7, and 14 d post‐induction, following *Salmonella* injection at day 3 and L‐arabinose induction at day 0. The low streptavidin concentration in the blood on day 1 suggests that streptavidin secretion occurred locally within the tumor and was effectively retained in the tumor microenvironment. By day 3, the increase in circulating streptavidin indicates a gradual release from the tumor into systemic circulation, a release profile that aligns with the sustained retention of biotin‐Cy7 in the tumor for up to 3 days (Figure 3f). Furthermore, the complete clearance of streptavidin from systemic circulation after day 7 demonstrates that the SAS system does not induce long‐term cytotoxic effects, reinforcing its biosafety and minimal impact on overall viability.

These results validate the imaging data by confirming the efficient clearance of streptavidin within a clinically relevant timeframe, minimizing systemic accumulation concerns, while also demonstrating the SAS system's favorable biosafety profile with controlled clearance dynamics, tumor‐specific targeting, and minimal systemic toxicity, reinforcing its potential for safe and effective clinical translation in fluorescence‐guided cancer surgery.

### 2.8 Clinical Translation Potential and Future Considerations

FGS remains an emerging field with limited clinical adoption, highlighting the strong potential for SAS‐based contrast agents in clinical translation. Unlike conventional targeted contrast agents that require tumor‐specific modifications, the SAS system offers a tumor‐agnostic approach, demonstrating its applicability across multiple cancer types. Its effectiveness was validated in CT26 and B16F10 tumor models, where bacterial selective colonization and prolonged fluorescence retention mirrored those observed in 4T1 tumors (Figures S6 and S9, Supporting Information).^[^ ^45^, ^46^ ^]^

Comparative analysis with conventional contrast agents further emphasizes the advantages of the SAS system. During average surgical durations (3–4 h for breast cancer,^[^ ^35^ ^]^ 3–5 h for colon cancer,^[^ ^47^ ^]^ and 2–4 h for melanoma^[^ ^48^ ^]^), conventional agents exhibited significant fluorescence loss, with 87% in breast cancer, 78% in colon cancer, and 94% in melanoma. In contrast, the SAS‐based contrast agent retained 79%, 100%, and 67% of fluorescence in these respective cancer models, ensuring stable imaging throughout surgery (Table S2, Supporting Information). Furthermore, direct comparison with FDA‐approved indocyanine green (ICG) demonstrated that the SAS system maintains comparable clearance dynamics while offering superior tumor retention and selectivity. In vivo administration of ICG in 4T1, CT26, and B16F10 tumors revealed clearance times of 86.3, 80.26, and 98.96 min, respectively, closely aligning with the clearance profiles of biotin‐Cy7 in control cases (72.58 min for 4T1, 167.44 min for CT26, and 63.45 min for B16F10) (Figure S9g,h and Table S3, Supporting Information). These comparable release profiles suggest that the SAS‐based contrast agent can integrate seamlessly into existing clinical workflows while enhancing tumor imaging efficiency. Together, these findings highlight the strong clinical potential of the SAS system as a reliable and versatile contrast agent for FGS, facilitating its transition toward clinical application.

Beyond preclinical validation, the clinical translation of bacteria‐based tumor imaging and therapy is gaining momentum, with engineered bacterial strains like *Salmonella typhimurium* (VNP20009) and SYNB1891 already in clinical trials (NCT04167137).^[^ ^49^ ^]^ The FDA‐approved Bacillus Calmette–Guérin (BCG) therapy for bladder cancer further demonstrates the feasibility of bacterial‐based treatments. Moreover, research on engineering bacteria for tumor targeting has been actively advancing,^[^ ^50^ ^]^ leading to ongoing clinical studies exploring their therapeutic applications.^[^ ^51^, ^52^ ^]^ However, several hurdles must be addressed before clinical implementation, including regulatory challenges, biosafety concerns, and host immune response modulation.^[^ ^53^, ^54^ ^]^ To mitigate these risks, extensive research has been conducted on genetic attenuation, controlled regulatory mechanisms, and biosafety evaluations. This study also demonstrated efficient bacterial clearance and minimal systemic toxicity, supporting the safety of bacterial‐based imaging approaches.

Advancements in synthetic biology and bioengineering are further expanding the potential of bacterial‐based imaging. Genetic modifications are being developed to enhance tumor targeting, reduce virulence, and improve overall safety profiles.^[^ ^50^ ^]^ Additionally, the SAS system's adaptability suggests potential integration with other imaging modalities, such as NIR‐II fluorescence imaging, which offers advantages like deeper tissue penetration and improved signal‐to‐noise ratios.^[^ ^55^ ^]^ Given its tumor‐selective targeting, the SAS system could also be engineered to deliver NIR‐II fluorophores, broadening its application in fluorescence‐guided surgery. Furthermore, although the present study focused on delineating margins of primary tumors for curative surgical resection, the detection of metastatic lesions–particularly small, disseminated foci–remains an unmet clinical need where bacterial imaging agents may also prove valuable. Expanding SAS‐based imaging to metastasis‐targeted applications represents an important direction for future research, especially for cancers where accurate localization of secondary lesions is essential for surgical or systemic intervention.These findings collectively support the clinical viability of the SAS‐based contrast system and emphasize the need for continued development to facilitate its transition into clinical oncology.

## 3 Conclusion

This study developed an SAS‐based contrast agent for fluorescence image‐guided tumor resection surgery, demonstrating its ability to enhance intra‐tumoral fluorescent signal retention, improve tumor‐selective imaging, and provide superior contrast efficiency. By leveraging intratumoral penetration, SAS enabled enhanced 3D tumor visualization, overcoming the limitations of conventional contrast agents. The system was validated in fluorescence image‐guided tumor resection surgery, accurately delineating tumor margins while minimizing positive margins. Long‐term biosafety evaluations confirmed minimal systemic toxicity and efficient bacterial clearance, reinforcing its safety for potential clinical applications. Furthermore, the SAS system demonstrated broad applicability across different tumor models, with fluorescence retention outperforming conventional contrast agents, suggesting its potential for various solid tumors beyond those tested. While factors like tissue density and the blood–brain barrier may influence performance in specific cancers, existing studies support the feasibility of bacterial therapies in overcoming these challenges. In validating the tumor‐targeting and contrast‐enhancing capabilities of the SAS platform, its potential for expansion into bacterial‐mediated tumor‐targeting (BMTT) applications becomes evident, as the inherent immune‐modulatory properties of bacteria provide a compelling basis for future therapeutic integration.Further investigations will be necessary to optimize SAS for a wider range of tumor types and enhance its clinical utility. These findings highlight SAS as a promising platform for precision surgical imaging, paving the way for its integration into clinical fluorescence‐guided surgery.

## 4 Experimental Section

### Bacterial Strains and Culture Conditions

The *Salmonella* strain BRD509 (aroA aroD‐, mutant of SL1344) was used,^[^ ^56^ ^]^ as well as a strain with asd and rcsB deletions (aroA aroD asd rcsB‐ of SL1344) (Table S1, Supporting Information). Thankfully obtained BRD509 and BRD509 asd– strains from Professor In‐Soo Lee at Hannam University. The rcsB gene was deleted from strain #178 using rcsB‐pkd13‐For and rcsB‐pkd13‐Re primers via the lambda‐mediated targeted mutagenesis method,^[^ ^57^ ^]^ resulting in the completion of the aroA aroD rcsB *Salmonella* strain (#245) (Table S4, Supporting Information). P22 phage (strain construction utilized either P22‐mediated generalized transduction)^[^ ^58^ ^]^ was employed to transfer rcsB from strain #245 to strain #177, resulting in the creation of strain #1237 (Table S1, Supporting Information). Strain #1237, containing flgM‐streptavidin, was confirmed as flgM‐streptavidin‐flhDC‐pBAD18‐asd+ / #1237 (aroA aroD asd rcsB‐) to yield strain #1594. All DNA manipulations were performed using the *E. coli* strain DH5a. The plasmids were transferred into *S. typhimurium* SF586 by electroporation and followed by transformation into the strain.

### Constructing a Plasmid for Streptavidin Secretion via the Flagellar Secretion System

flgM‐streptavidin was designed by fusing the flgM protein with engineered Monomeric Streptavidin (Figure S1a, Supporting Information). This engineered monomeric streptavidin contains an N‐terminal 6xHis tag and a C‐terminal FLAG (DYKDDDDK) tag.^[^ ^59^ ^]^ The amino acid sequences of flgM and streptavidin are shown in Figure S1b (Supporting Information), and the codon‐optimized DNA sequence of this fusion protein was synthesized by Cosmogenetech (Seoul, Korea) as flgM‐engineered monomeric streptavidin gene (Figure S1c, Supporting Information).

A plasmid encoding recombinant streptavidin was constructed by ligating a DNA fragment encoding flgM‐streptavidin with pBAD18 after digestion with NheI (1241A; Takara, JAPAN) and SacI (1078A; Takara, JAPAN). The resulting plasmid was named flgM‐streptavidin. The coding sequences of flhDC together with the RBS were further cloned into flgM‐streptavidin following SacI and SalI (1080A, Takara, JAPAN) digestion. LT2 *Salmonella* was PCR‐amplified using flhD‐Sac1‐for and flhC‐Sal1‐re primers and then ligated into the restriction sites behind flgM‐streptavidin using enzymes (Table S3, Supporting Information).

The streptavidin‐associated *Salmonella* strains were cultured overnight at 37 °C and 250 rpm in LuriaBertani broth (LB; 1% w/v of tryptone, 1% w/v of NaCl, 0.5% w/v of yeast extract, supplemented with 100 µg/mL of ampicillin). The overnight culture was diluted 100‐fold into 50 mL LB and incubated at 37 °C and 250 rpm until reaching an OD_600_ of 0.4–0.6. Upon reaching the desired OD, the cultures were centrifuged for 2 min at 8200 rpm, and the supernatant was discarded. The bacterial pellet was then resuspended in 1 mL of culture per OD 1.0, ensuring a concentration of 4 x 10^8^ CFU per mL for experimental use.

### Measurement of Streptavidin Secretion in Bacteria by Western Blot Analysis

The strain #1237 containing the flgM‐streptavidin plasmid, also known as #1594, was grown in LB medium at 37 °C with vigorous aeration (200 rpm) for 1 h. After induction with 0.04% (v/v) L‐arabinose for flgM‐streptavidin expression, the bacteria were further cultured for 6 h. Bacterial cells were then harvested by centrifugation at 13,000 x g for 3 min. The resulting pellet was resuspended in 1 mL of 1×PBS, and the culture supernatant was filtered through a 0.2 µm pore size filter device. Each fraction was mixed with 0.2% sodium dodecyl sulfate (SDS) loading buffer containing β‐mercaptoethanol. Total cell lysates were prepared using centrifugation without separation. The whole‐cell lysates and each fraction were separated by 12% SDS‐polyacrylamide gel electrophoresis (PAGE) and transferred onto a nitrocellulose membrane (LC7034‐300; GenDEPOT) for Western blot analysis. The membrane was blocked at room temperature for 1 h with blocking buffer, followed by incubation with primary antibodies anti‐His tag (SB194b, SouthernBiotech), anti‐FLAG antibody (F3165, Sigma‐Aldrich, USA), and anti‐DnaK antibody (ab69617, abcam, UK) diluted 1/1000 at 4 °C overnight. After washing the membrane three times with Tris‐buffered saline with 0.1% (v/v) Tween‐20 (TBST), secondary antibodies anti‐mouse‐IgG (7076s, Cell Signaling, Danvers, MA, USA) and anti‐rabbit IgG antibody (for DnaK, 7074, Cell Signaling Technology) were used at a dilution of 1:5000 in TBST containing 2.5% skim milk at room temperature for 1 h. The membrane was then washed three times with TBST at 10‐min intervals, followed by detection of protein expression using chemiluminescent substrate (32209, Thermo Fisher Scientific) on an iBright CL1500 imaging system (Thermo Fisher Scientific).

### Cell Growth Assay and Western Blot Assay

Strain #1594 was grown overnight with shaking in LB medium, and the OD_600_ was adjusted to approximately 0.05. The culture was incubated at 37 °C and 250 rpm. After 1 h, two groups were formed: the uninduced group (green) and the group induced with 0.2% (w/v) L‐arabinose in di water (final concentration) (blue). The OD_600_ of each culture was measured hourly following induction for 1, 2, 3, 4, 5, and 6 h. At each time point, 1 mL of supernatant was concentrated, and Western blotting was performed using anti‐His tag antibodies to detect FlgM‐Streptavidin. The Western blotting procedure was the same as described in the section of “Measurement of Streptavidin Secretion in Bacteria by Western Blot Analysis” using anti‐His tag antibodies.

### ELISA Assay

Using the same method as in the section of “Cell Growth Assay and Western Blot Assay,” strain #1594 was divided into uninduced (non‐induction) and induced (induction with 0.2% (w/v) L‐arabinose in di water) groups. After 6 h, supernatants from each group were collected, and the amount of FlgM‐Streptavidin was measured using the His Tag ELISA Detection Kit (L00436, GenScript, USA).

### Motility Assay

Motility assays were conducted using soft agar tryptone plates (per liter: 10 g Bacto tryptone, 5 g NaCl, and 3 g Bacto agar). A bacterial colony was selected using a toothpick and inoculated into the soft agar, followed by incubation at 37 °C for approximately 4 h. If necessary, 0.2% (w/v) L‐arabinose was added for pBAD induction.

### HABA Assay

An experiment was conducted to verify the secretion performance of streptavidin through bacterial induction. At intervals of 1 h after the start of induction, a portion of the culture was separated, and centrifuged at 8200 rpm for 2 min to obtain the supernatant containing streptavidin. The principle of increasing absorbance by the binding of HABA (4'‐hydroxyazobenzene‐2‐carboxylic acid) to streptavidin present in the supernatant was utilized to design a method where no absorbance change occurs when streptavidin in the supernatant binds to biotin added artificially (Figure 2f). EZ‐Link Sulfo‐NHS‐Biotin (Thermo *Scientific* ^*TM*^, cat#21425) was prepared at a concentration of 1.65 mg/mL in ultrapure water (UP). In cases where biotin was added, the biotin solution and the supernatant were reacted for 30 min on a 500 rpm shaker at the same volume ratio. Subsequently, they were reacted with a 0.3 mM HABA solution (Thermo Scientific) at a volume ratio of 9:1 and the absorbance at 500 nm was measured. This was compared with the case where biotin was absent to verify the streptavidin secretion of SAS. Additionally, to quantify the amount of functionalized streptavidin on the bacterial surface, biotin (20µL of 0.1g/L) and Flamma 749 (Bioacts, Incheon) were added at each h of induction duration to induce bacterial binding. The ratio of bacteria bound to biotin‐Cy7 to the total number of bacteria was quantified through the presence or absence of fluorescent signals observed under a microscope (Zeiss AxioObserver Z1).

### In Vitro Streptavidin Associated Fluorescent Signal Retainment Validation Assay

Furthermore, we validated the enhancement of fluorescence signal maintenance within cancerous tissues using the proposed SAS system in an in vitro cancerous tissue mimicry experimental device employing porous agarose, as shown in Figure 3a. The bottom compartment of the confocal dish was solidified into a gel form. (Control; agarose 0.72% w/v, LB 14% v/v, PBS 14% v/v, SAS (−); agarose 0.72% w/v, SAS(4 x 10^7^) in LB 14% v/v, PBS 14% (v/v), SAS (+); agarose 0.72% w/v, SAS(4 x 10^7^) in LB 14% v/v, L‐arabinose 5.71% w/v). Then, biotin‐Cy7 (20µL of 0.1g/L) was added to each well overnight to allow diffusion into the cancerous tissue mimic platform at 37 °C. The following day, to mimic fluorescent dye drainage, PBS washing was performed hly while acquiring fluorescence images every 30 min at different depths of the agar (Z1 ∼ Z4 in Figure S3a, Supporting Information), and signal intensity (0 ∼ 255) was measured and analyzed using ImageJ (NIH, Bethesda, MD). The bottom Z1 of the agar was designed to represent the depth of the tumor as the necrotic area, progressing upwards to mimic the surface of the tumor. Fluorescence images were acquired through a microscope (Zeiss AxioObserver Z1).

### Mammalian Cell Culture (4T1, CT26, and B16F10)

Three cell lines ‐ mouse breast cancer cells 4T1 (ATCC CRL‐2539), colon cancer cells CT26 (ATCC CRL‐2638), and melanoma cells B16F10 (ATCC CRL‐6475)) were used. Cells were cultured in T75 flasks with RPMI 1640 (Gibco; for 4T1, CT26) and DMEM (Gibco; for B16F10) containing 10% v/v Fetal Bovine Serum (FBS; Gibco) and 1% v/v penicillin‐spectinomycin (Gibco) at 37 °C in a 5% CO_2_ environment. When the cells reached 80–90% confluency in the flask, they were detached using 0.25% trypsin‐EDTA (Gibco) 3 mL, counted using a hemocytometer, and diluted to the appropriate concentration for the respective experiments.

### Animal Study

All animal experiments were conducted in strict accordance with the guidelines of the Institutional Animal Care and Use Committee (IACUC, approval number: KIST‐IACUC‐2023‐026‐1). Female 5‐week‐old BALB/c mice (for 4T1 and CT26 tumors) and C57BL/6 mice (for B16F10 tumors) were obtained from Orientbio, Sungnam, and allowed a week for environmental adaptation. Each mouse was then subcutaneously injected with 1 x 10^6^ cancer cells in 30 µL into the left thigh. When the tumor size reached 300–500 mm^3^, the mice were anesthetized, and 5 x 10^7^ CFU of the SAS strain in 100 µL was administered intravenously via the tail vein. After 24 h, streptavidin secretion was induced by intraperitoneally injecting 200 µL of 40% (w/v) L‐arabinose in di water. In 2 h, 20 µL of biotin‐Cy7 at 0.1g/L was injected subcutaneously around the tumor. Tissues, including the tumor, surrounding skin, liver, spleen, and kidney, were harvested at 2, 6, and 12 h post‐injection following euthanasia of the mice. To confirm bacterial colonization in hypoxic regions, SAS with GFP plasmid strain was injected into 4T1 tumors, and after 24 h, the mice were euthanized, tumors were extracted, sectioned, and stained with Nucblue (Thermofisher). Fluorescence microscopy (Zeiss AxioObserver Z1) was used to capture images of the slices.

### Fabrication of the Macroscope and Fluorescence Imaging

To confirm the fluorescently labeled lesion area during surgery, fluorescence imaging devices such as IVIS are not feasible due to spatial constraints. Recently, surgical microscopes equipped with fluorescence functionality have been developed, but they are not specialized for the fluorescent contrast agent used in this study and are very expensive.^[^ ^60^, ^61^ ^]^ Therefore, a macroscope was custom‐made for this study. This macroscope is designed to capture a 10 cm X 10 cm area from 30 cm above the lesion, allowing visualization of both three‐dimensional visible light images and fluorescence images simultaneously, enabling precise delineation of lesion boundaries by the surgeon (Figure S4a, Supporting Information). For fluorescence imaging, a 780 nm longpass filter was attached to a monochrome camera, and a 700 nm LED collimated light source was used as the excitation light. Figure S4a (Supporting Information) illustrates the configuration of the macroscope. The image transformation matrix between the left/right RGB camera and the monochrome camera is obtained in advance using stereo calibration methods for each pair. Based on this transformation matrix, the fluorescence images obtained from the monochrome camera are overlaid onto the RGB images for visualization. The left/right RGB images can be viewed stereoscopically by the surgeon through a head‐mounted display (HMD) or visualized on a three‐dimensional monitor. Figure S4b (Supporting Information) shows the result of overlaying the fluorescence image onto the RGB image.

The overall trends observed in fluorescent signal intensity analyzed using the macroscope were similar to those obtained from IVIS imaging. However, when comparing the 12 h tumor images in Figure 4c obtained from IVIS and macroscope, a greater decrease in fluorescent signal intensity was observed with the macroscope. This discrepancy is attributed to differences in sensitivity according to the depth under the surface between IVIS and macroscope imaging. IVIS demonstrated better recognition of fluorescent signals from deeper locations compared to the macroscope (Figure S4c–e, Supporting Information). Fluorescent agar was prepared by adding 100 µL of 1 g/L biotin‐Cy7 to 900 µL of 1.5% agar. After solidifying 100 µL of fluorescent agar in a cuvette, an additional 0–900 µL of 1.5% agar was layered on top and allowed to solidify before fluorescence imaging was performed.

### Fluorescence Imaging for Intraoperative Tumor Localization

Fluorescence imaging to assess tumor contrast efficiency during surgery was conducted using the IVIS (Caliper, USA) filter set: λ_*exc*_ = 745 nm, λ_*emi*_ = 800 nm. Tumor, skin tissue at the site of fluorescence injection, liver, spleen, and kidneys were captured under the same imaging conditions, and fluorescent signal intensity was analyzed. The same method was applied to capture images of organs using a Macroscope available for use during surgery. To verify the distribution of fluorescent dye within the tumor, the tumor was sectioned perpendicular to the injection site into five equal parts, and fluorescence images of the sections were captured. TBR quantified by dividing the fluorescent signal intensity of the tumor by the fluorescent signal intensity of the skin tissue at the injection site.

### Time Course Fluorescent Signal Distribution

Fluorescence imaging to observe changes in the residence time of the fluorescent dye depending on the presence of SAS was conducted using the IVIS (Caliper, USA) filter set: λ_*exc*_ = 745 nm, λ_*emi*_ = 800 nm. Time‐dependent changes in fluorescent signal intensity were analyzed from the time of fluorescent dye injection via peritumoral injection until 3 days thereafter.

### Tumor Colonized Bacteria Counting

To determine the number of bacteria remaining in the three types of tumor and major organs, a portion of excised tumor and major organs was sectioned, weighed, and homogenized in 1 mL of PBS. The samples were then spread onto 1.5% LB agar plates (with 100 µg/mL of ampicillin) and incubated overnight at 37 °C to count colony‐forming units (CFU) and measure the concentration of bacteria present in the tumor and organs.

### SAS Mediated Fluorescence Image Guided Tumor Dissecting Surgery

To assess the effectiveness of tumor imaging, fluorescence image‐guided surgery was performed using SAS. For the orthotopic tumor model, 5‐week‐old female Balb/c mice (OrientBio, Sungnam) were acclimatized for a week. Tumor models were established by anesthetizing the mice via isoflurane inhalation, followed by subcutaneous injection of 1 x 10^6^ 4T1 cells suspended in 20 µL of RPMI 1640 into the mammary fat pad. When the tumor size reached 300–500 mm^3^, 5 × 10^7^ CFU of the SAS strain in 100 µL was administered intravenously via the tail vein. After 24 hours, streptavidin secretion was induced by intraperitoneal injection of 200 µL of 40% (w/v) L‐arabinose. Two hours later, 20 µL of 0.1 g/L biotin‐Cy7 was subcutaneously injected around the tumor. Tumor resection was performed 6 h after the injection of biotin‐Cy7 to align with clinically relevant surgical durations, which typically range from 3 to 4 h,^[^ ^27^, ^62^ ^]^ including preparation time. This selection ensures that the fluorescent signal remains stable throughout the procedure while also accounting for potential variations in surgical length due to tumor morphology and patient conditions. The procedure began with the removal of the abdominal skin under white light, while the visible fluorescent signal was continuously monitored in real‐time under macroscope (Movie S1, Supporting Information). Tumor was excised based on the fluorescence‐guided locations. Resection was continued until no residualfluorescent signal could be detected, ensuring the complete removal of the tumor. Surrounding tissues showing no fluorescent signal were further excised (3^rd^ section in Figure 5b; Figure S7, Supporting Information) to confirm the absence of residual tumor cells.

The excised tissues were fixed in 4% paraformaldehyde (PFA) and subsequently underwent hematoxylin and eosin (H&E) staining for histological evaluation. All stained samples were independently reviewed by pathologist Professor Jin‐Man Kim, who was blinded to the clinical information.

### Comprehensive Biosafety and Pharmacokinetic Evaluation—Evaluation

**Figure 6:**
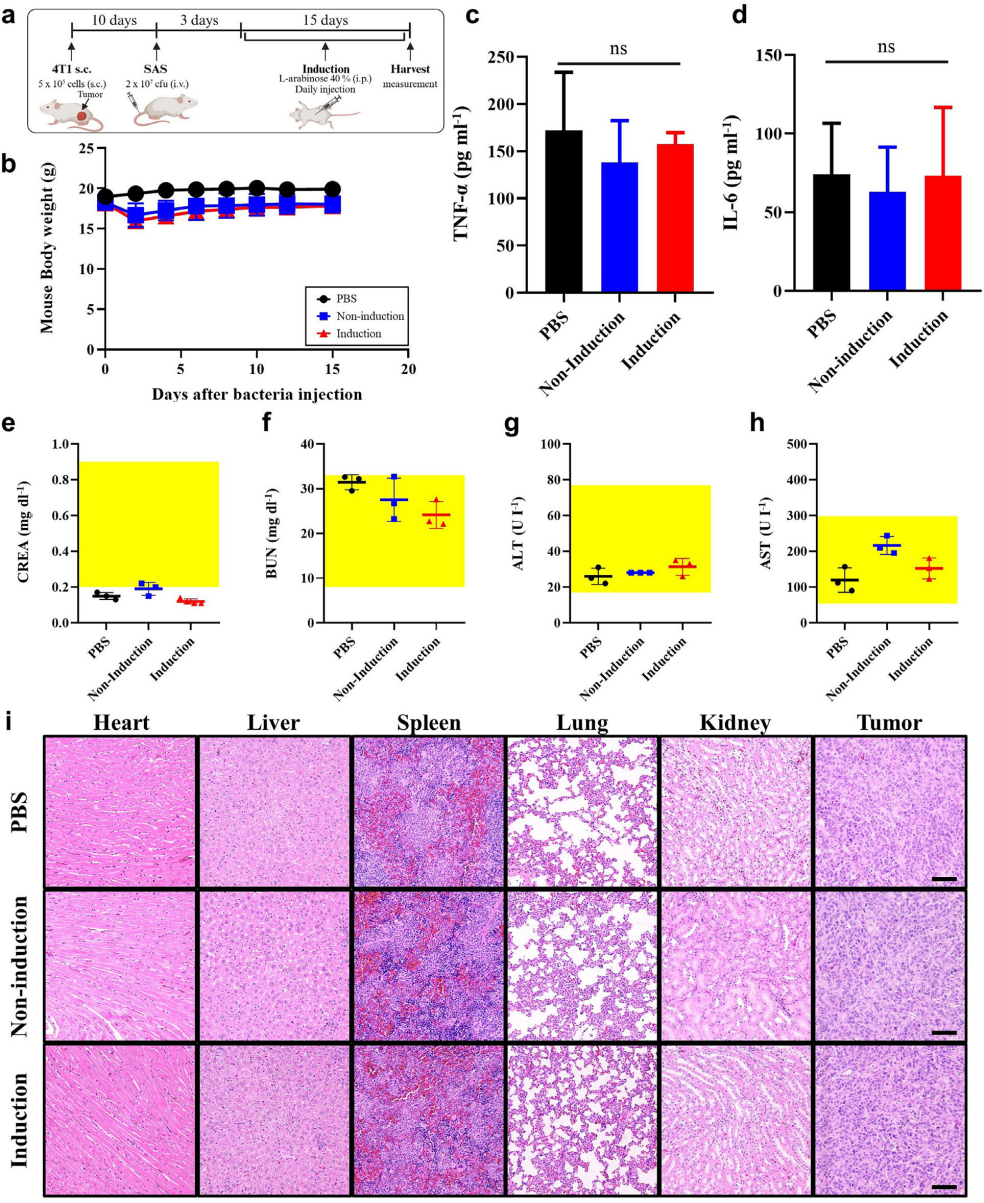
Streptavidin‐secreting *Salmonella* demonstrates biological safety in vivo. a) Experimental design in mice bearing 4T1 tumors (*n* = 3). *Salmonella* secreting streptavidin (2 x 10^7^ CFU) was intravenously injected, and observations were made on day 15. b) Body weight changes in mice after Salmonella administration. c) TNF‐α levels measured in serum on day 15 post‐administration. d) IL‐6 levels measured in serum on day 15 post‐administration. e–h) Biochemical indicators, including CREA, BUN, ALT, and AST, measured in serum on day 15 post‐administration. The yellow‐shaded areas represent the average normal range (*n* = 3). Data are presented as mean ± SEM. e) 0.2–0.9 mg/dL. f) 8–33 mg/dL. g) 17–77 U/I. h) 54–298 U/I. i) Representative histological images of various tissues from tumor‐bearing mice treated with Salmonella. Tissue sections (heart, liver, spleen, lung, kidney, and tumor) were prepared on day 15 after Salmonella treatment and stained with H&E. (Scale bar = 50 µm) Statistical analyses were performed using paired *t*‐tests. (*p < 0.05).

As shown in **Figure** **6**a, when 4T1 tumors reached 150 mm^3^, SAS bacteria (2 × 10^7^ CFU) were administered, followed by daily L‐arabinose injections (200 µL of 40% (w/v)) starting three days post‐inoculation. After 15 days, blood samples were collected from each group and centrifuged at 8,000 rpm for 10 minutes at 4 °C to obtain serum. For the measurement of inflammatory cytokines, mouse TNF‐α (Abcam, Cat#Ab208348) and IL‐6 (Abcam, Cat#Ab222503) ELISA kits were purchased from Abcam. Biochemical markers in serum, including alanine aminotransferase (ALT), aspartate aminotransferase (AST), blood urea nitrogen (BUN), and creatinine (CREA), were analyzed using the respective assay kits.

### Comprehensive Biosafety and Pharmacokinetic Evaluation—Immunohistochemical Analysis

For immunohistochemical analysis, tissue samples from the heart, liver, spleen, lung, kidney, and tumor were collected 15 days after *Salmonella* treatment and embedded in 4 µm‐thick paraffin sections for H&E staining to assess histopathological changes.

### Comprehensive Biosafety and Pharmacokinetic Evaluation—Measurement of Streptavidin in blood by ELISA

To measure streptavidin levels in blood, 4T1 tumor cells (1 × 10^6^) were subcutaneously injected into the left thigh of mice. When tumors reached 300–500 mm^3^, SAS bacteria (5 × 10^7^ CFU in 100 µL) were administered intravenously via the tail vein. Three days later, streptavidin secretion was induced by intraperitoneal injection of 200 µL of 40% (w/v) L‐arabinose. Blood samples were collected at 1, 3, 7, and 14 days, centrifuged at 2,000 g for 10 minutes using a serum separation tube to obtain serum and streptavidin levels were quantified using the Streptavidin ELISA Detection Kit (AKR‐5186, USA).

### Statistical Analysis

All statistical analyses were performed using Origin software. Paired t‐tests were conducted to compare experimental groups, with statistical significance determined at *p* < 0.05. Data are presented as mean ± standard deviation (SD), and all experiments were conducted with at least three independent replicates to ensure reproducibility.

## Conflict of Interest

The authors declare no conflict of interest.

## Supporting information

Supporting Information

Supplemental Movie 1

## Data Availability

The data that support the findings of this study are available from the corresponding author upon reasonable request.

## References

[1] F. Lyon , global Cancer Burden Growing, Amidst Mounting Need For Services, https://www.who.int/news/item/01‐02‐2024‐global‐cancer‐burden‐growing–amidst‐mounting‐need‐for‐services/, “[Online; World Health Organization News]” 2024. PMC11115397 38438207

[2] R. K. Orosco , V. J. Tapia , J. A. Califano , B. Clary , E. E. Cohen , C. Kane , S. M. Lippman , K. Messer , A. Molinolo , J. D. Murphy , V. J. Tapia , J. A. Califano , B. Clary , E. E. W. Cohen , C. Kane , S. M. Lippman , K. Messer , A. Molinolo , J. D. Murphy , J. Pang , A. Sacco , K. R. Tringale , A. Wallace , Q. T. Nguyen , Sci. Rep. 2018, 8, 5686. 29632347 10.1038/s41598-018-23403-5 PMC5890246

[3] J. S. D. Mieog , F. B. Achterberg , A. Zlitni , M. Hutteman , J. Burggraaf , R.‐J. Swijnenburg , S. Gioux , A. L. Vahrmeijer , Nat. Rev. Clin. Oncol. 2022, 19, 9. 34493858 10.1038/s41571-021-00548-3

[4] K. Wang , C. Chi , Z. Hu , M. Liu , H. Hui , W. Shang , D. Peng , S. Zhang , J. Ye , H. Liu , et al., Engineering 2015, 1, 309.

[5] H. L. Stewart , D. J. Birch , Methods and Applications in Fluorescence 2021, 9, 042002. 10.1088/2050-6120/ac1dbb 34399409

[6] K. Wang , Y. Du , Z. Zhang , K. He , Z. Cheng , L. Yin , D. Dong , C. Li , W. Li , Z. Hu , C. Zhang , H. Hui , C. Chi , J. Tian , Nat. Rev. Bioeng. 2023, 1, 161.

[7] M. D. Farwell , D. A. Pryma , D. A. Mankoff , Cancer 2014, 120, 3433. 24947987 10.1002/cncr.28860

[8] M. N. van Oosterom , P. Meershoek , M. M. Welling , F. Pinto , P. Matthies , H. Simon , T. Wendler , N. Navab , C. J. van de Velde , H. G. van der Poel , et al., IEEE Trans. Med. Imaging 2019, 39, 226. 31247546 10.1109/TMI.2019.2924254

[9] S. B. Mondal , S. Gao , N. Zhu , R. Liang , V. Gruev , S. Achilefu , Adv. Cancer Res. 2014, 124, 171. 25287689 10.1016/B978-0-12-411638-2.00005-7 PMC4245053

[10] S. Hernot , L. van Manen , P. Debie , J. S. D. Mieog , A. L. Vahrmeijer , Lancet Oncol. 2019, 20, e354. 31267970 10.1016/S1470-2045(19)30317-1

[11] C. Wang , Z. Wang , T. Zhao , Y. Li , G. Huang , B. D. Sumer , J. Gao , Biomaterials 2018, 157, 62. 29245052 10.1016/j.biomaterials.2017.12.002 PMC6502237

[12] S. Zhu , R. Tian , A. L. Antaris , X. Chen , H. Dai , Adv. Mater. 2019, 31, 1900321. 10.1002/adma.201900321 PMC6555689 31025403

[13] W. Stummer , E. S. Molina , Neurosurg. Clin. 2017, 28, 569. 10.1016/j.nec.2017.05.009 28917285

[14] K. Sano , T. Temma , T. Azuma , R. Nakai , M. Narazaki , Y. Kuge , H. Saji , Mol. Imaging Biol. 2011, 13, 1196. 21140232 10.1007/s11307-010-0463-1

[15] A. B. Ariffin , P. F. Forde , S. Jahangeer , D. M. Soden , J. Hinchion , Cancer Res. 2014, 74, 2655. 24778418 10.1158/0008-5472.CAN-13-3696

[16] S. Suh , A. Jo , M. A. Traore , Y. Zhan , S. L. Coutermarsh‐Ott , V. M. Ringel‐Scaia , I. C. Allen , R. M. Davis , B. Behkam , Adv. Sci. 2019, 6, 1801309. 10.1002/advs.201801309 PMC6364498 30775227

[17] M. T.‐Q. Duong , Y. Qin , S.‐H. You , J.‐J. Min , Exp. Mol. Med. 2019, 51, 1. 10.1038/s12276-019-0297-0 PMC6906302 31827064

[18] V. H. Nguyen , H.‐S. Kim , J.‐M. Ha , Y. Hong , H. E. Choy , J.‐J. Min , Cancer Res. 2010, 70, 18. 20028866 10.1158/0008-5472.CAN-09-3453

[19] Z. Mi , Z.‐C. Feng , C. Li , X. Yang , M.‐T. Ma , P.‐F. Rong , J. Cancer 2019, 10, 4765. 31598148 10.7150/jca.32650 PMC6775532

[20] S. Zhou , C. Gravekamp , D. Bermudes , K. Liu , Nat. Rev. Cancer 2018, 18, 727. 30405213 10.1038/s41568-018-0070-z PMC6902869

[21] F. Badie , M. Ghandali , S. A. Tabatabaei , M. Safari , A. Khorshidi , M. Shayestehpour , M. Mahjoubin‐Tehran , K. Morshedi , A. Jalili , V. Tajiknia , M. R. Hamblin , H. Mirzaei , Front Oncol 2021, 11, 624759. 33738260 10.3389/fonc.2021.624759 PMC7960920

[22] D. Chandra , B. C. Selvanesan , Z. Yuan , S. K. Libutti , W. Koba , A. Beck , K. Zhu , A. Casadevall , E. Dadachova , C. Gravekamp , Oncotarget 2017, 8, 20729. 28186976 10.18632/oncotarget.15117 PMC5400540

[23] P. Sarotra , B. Medhi , Current Strategies in Cancer Gene Therapy 2016, 111.

[24] N. Nair , T. Kasai , M. Seno , AntiCancer Res. 2014, 34, 6289. 25368227

[25] X. Huang , J. Pan , F. Xu , B. Shao , Y. Wang , X. Guo , S. Zhou , Adv. Sci. 2021, 8, 2003572. 10.1002/advs.202003572 PMC8025040 33854892

[26] C. Hoogstins , J. J. Burggraaf , M. Koller , H. Handgraaf , L. Boogerd , G. van Dam , A. Vahrmeijer , J. Burggraaf , Mol. Imaging Biol. 2019, 21, 11. 29845427 10.1007/s11307-018-1220-0

[27] S. Van Keulen , M. Hom , H. White , E. L. Rosenthal , F. M. Baik , Mol. Imaging Biol. 2023, 25, 36. 36123445 10.1007/s11307-022-01772-8 PMC9971137

[28] F. Azari , G. Kennedy , E. Bernstein , J. Delikatny , J. Y. Lee , J. Kucharczuk , P. S. Low , S. Singhal , Mol. Imaging Biol. 2023, 25, 85. 34101106 10.1007/s11307-021-01618-9 PMC8651846

[29] S. Guo , I. Alshamy , K. T. Hughes , F. F. Chevance , J. Bacteriol. 2014, 196, 2333. 24706743 10.1128/JB.01572-14 PMC4054164

[30] H. M. Singer , M. Erhardt , A. M. Steiner , M.‐M. Zhang , D. Yoshikami , G. Bulaj , B. M. Olivera , K. T. Hughes , MBio 2012, 3, 10. 10.1128/mBio.00115-12 PMC3372961 22647788

[31] L.‐M. Guzman , D. Belin , M. J. Carson , J. Beckwith , J. Bacteriol. 1995, 177, 4121. 7608087 10.1128/jb.177.14.4121-4130.1995 PMC177145

[32] H. Loessner , A. Endmann , S. Leschner , K. Westphal , M. Rohde , T. Miloud , G. Hämmerling , K. Neuhaus , S. Weiss , Cell. Microbiol. 2007, 9, 1529. 17298393 10.1111/j.1462-5822.2007.00890.x

[33] J. E. Galán , K. Nakayama , R. Curtiss III , Gene 1990, 94, 29. 2227450 10.1016/0378-1119(90)90464-3

[34] M. Choi , H. W. Choi , H. E. Kim , J. Hahn , Y. J. Choi , Food Hydrocolloids1 2023, 145, 109043.

[35] J. C. Boughey , F. Goravanchi , R. N. Parris , S. S. Kee , A. M. Kowalski , J. C. Frenzel , I. Bedrosian , F. Meric‐Bernstam , K. K. Hunt , F. C. Ames , H. M. Kuerer , A. Lucci , Am. J. Surg. 2009, 198, 720. 19427625 10.1016/j.amjsurg.2008.11.043 PMC5842353

[36] S. J. Lee , Y. Y. Choi , C. Kim , M. S. Chung , J. Korean Med. Sci. 2017, 32, 1031. 28480663 10.3346/jkms.2017.32.6.1031 PMC5426239

[37] C. Clinic , breast Cancer Surgery, https://my.clevelandclinic.org/health/treatments/8338‐breast‐cancer‐surgery/, 2023, [Online; Cleveland Clinic Health Library].

[38] J. Lustgarten , how Long Is Surgery To Remove A Brain Tumor?, https://www.neurosurgeonsofnewjersey.com/blog/how‐long‐is‐surgery‐to‐remove‐a‐brain‐tumor/, 2019, [Online; Neurosurgeons of New Jersey].

[39] J. Cafasso , your Guide To Colon Cancer Surgery: Key Terms And Faqs, https://www.healthline.com/health/colorectal‐cancer/colon‐cancer‐surgery#fa‐qs/, 2022, [Online; healthline Medically reviewed].

[40] W. C. Conway , peritonectomy For Mesothelioma, https://www.asbestos.com/treatment/surgery/peritonectomy/, 2024, [Online; Asbestos.com Surgery Procedures].

[41] G. bladder cancer institute, what To Expect Before And After Surgery, https://www.hopkinsmedicine.org/greenberg‐bladder‐cancer‐institute/bladder‐cancer‐treatment/before‐after‐surgery/, [Online; Johns Hopkins Medicine Greenburg bladder cancer institute].

[42] O. U. Akakuru , Z. Zhang , M. Z. Iqbal , C. Zhu , Y. Zhang , A. Wu , Acta Pharmaceutica Sinica B 2022, 12, 2640. 35755279 10.1016/j.apsb.2022.02.016 PMC9214073

[43] Y. Fu , F. Ye , X. Zhang , Y. He , X. Li , Y. Tang , J. Wang , D. Gao , ACS nano 2022, 16, 18376. 36355037 10.1021/acsnano.2c06356

[44] R. Matsui , N. Inaki , T. Tsuji , T. Fukunaga , Cancers 2024, 16, 833. 38398224 10.3390/cancers16040833 PMC10886510

[45] H.‐J. Cho , S.‐J. Park , Y.‐S. Lee , S. Kim , J. Controlled Release 2019, 300, 73. 10.1016/j.jconrel.2019.02.043 30831135

[46] S. D. Steichen , M. Caldorera‐Moore , N. A. Peppas , Eur. J. Pharm. Sci. 2013, 48, 416. 23262059 10.1016/j.ejps.2012.12.006 PMC3619023

[47] M. B. Bailey , D. L. Davenport , H. D. Vargas , B. M. Evers , S. P. McKenzie , Dis. Colon Rectum 2014, 57, 616. 24819102 10.1097/DCR.0000000000000114

[48] B. van Etten , J. de Wilt , F. Brunstein , A. Eggermont , C. Verhoef , Eur. J. Surg. Oncol. (EJSO) 2009, 35, 539. 18760560 10.1016/j.ejso.2008.07.004

[49] J. F. Toso , V. J. Gill , P. Hwu , F. M. Marincola , N. P. Restifo , D. J. Schwartzentruber , R. M. Sherry , S. L. Topalian , J. C. Yang , F. Stock , L. J. Freezer , K. E. Morton , C. Seipp , L. Haworth , S. Mavroukakis , D. White , S. MacDonald , J. Mao , M. Sznol , S. A. Rosenberg , Int. J. Clin. Oncol. 2002, 20, 142. 10.1200/JCO.2002.20.1.142 PMC2064865 11773163

[50] C. R. Gurbatri , N. Arpaia , T. Danino , Science 2022, 378, 858. 36423303 10.1126/science.add9667 PMC10584033

[51] B. F.‐L. Sieow , K. S. Wun , W. P. Yong , I. Y. Hwang , M. W. Chang , Trends Cancer 2021, 7, 447. 33303401 10.1016/j.trecan.2020.11.004

[52] S.‐Y. Kwon , H. Thi‐Thu Ngo , J. Son , Y. Hong , J.‐J. Min , Nat. Rev. Clin. Oncol. 2024, 21, 569. 38840029 10.1038/s41571-024-00908-9

[53] W. Zhen , Z. Wang , Q. Wang , W. Sun , R. Wang , W. Zhang , Y. Zhang , W. Qin , B. Li , Q. Wang , et al., Imeta 2024, 3, e197. 38898992 10.1002/imt2.197 PMC11183164

[54] Z. Wang , W. Sun , R. Hua , Y. Wang , Y. Li , H. Zhang , Int J Oral Sci 2024, 16, 24. 38472176 10.1038/s41368-024-00282-3 PMC10933493

[55] S. Roy , N. Bag , S. Bardhan , I. Hasan , B. Guo , Adv. Drug. Deliv. Rev. 2023, 197, 114821. 37037263 10.1016/j.addr.2023.114821

[56] R. Strugnell , G. Dougan , S. Chatfield , I. Charles , N. Fairweather , J. Tite , J. L. Li , J. Beesley , M. Roberts , Infect. Immun. 1992, 60, 3994. 1398911 10.1128/iai.60.10.3994-4002.1992 PMC257428

[57] K. A. Datsenko , B. L. Wanner , Proc. Natl Acad. Sci. 2000, 97, 6640. 10829079 10.1073/pnas.120163297 PMC18686

[58] J. E. Karlinsey , Methods Enzymol. 2007, 421, 199. 17352924 10.1016/S0076-6879(06)21016-4

[59] K. H. Lim , H. Huang , A. Pralle , S. Park , Biotechnol. Bioeng. 2013, 110, 57. 22806584 10.1002/bit.24605

[60] T. Sato , K. Suzuki , J. Sakuma , N. Takatsu , Y. Kojima , T. Sugano , K. Saito , Acta Neurochir 2015, 157, 1295. 26148906 10.1007/s00701-015-2481-x

[61] J. R. Watson , C. F. Gainer , N. Martirosyan , J. Skoch , G. M. Lemole Jr , R. Anton , M. Romanowski , J Biomed Opt 2015, 20, 106002. 26440760 10.1117/1.JBO.20.10.106002 PMC4881285

[62] M. Morneau , J. Boulanger , P. Charlebois , J.‐F. Latulippe , R. Lougnarath , C. Thibault , N. Gervais , Can. J. Surg. 2013, 56, 297. 24067514 10.1503/cjs.005512 PMC3788008

