# Supplementary material for: Engineering Bacterial Secretion Systems for Enhanced Tumor Imaging and Surgical Guidance
Source: Adv Mater. 2025 May 21;37(34):2504389. doi: 10.1002/adma.202504389 (PMC12392856; doi:10.1002/adma.202504389)
Supplement: Supplementary file 1 — Supporting Information [file ADMA-37-2504389-s001.pdf]

ADVANCED MATERIALS

Supporting Information

for *Adv. Mater.*, DOI 10.1002/adma.202504389

Engineering Bacterial Secretion Systems for Enhanced Tumor Imaging and Surgical Guidance

Dohee Lee, Heung Jin Jeon, Dohyub Jang, Deukhee Lee, Solbi Kim, Minju Han, Sharon Jiyoon Jung, Jung-Hyun Lee, Jia Choi, Dong Ha Kim, Dong June Ahn, Keri Kim, Sehoon Kim, Hyo-Jin Lee* and SeungBeum Suh**

Supplementary Information

Engineering Bacterial Secretion Systems for Enhanced Tumor Imaging and Surgical Guidance

Dohee Lee†[1,4] Heung Jin Jeon†[2] Dohyub Jang†[3,5] Deukhee Lee[1] Solbi Kim[2] Minju Han[2] Sharon Jiyoon Jung[6] Jung-Hyun Lee[4] Jia Choi[3,7] Dong Ha Kim[9], Dong June Ahn[4,5] Keri Kim[1] Sehoon Kim*[3,7] Hyo-Jin Lee*[2] SeungBeum Suh*[1,8]

1. Bionics Research Center, Korea Institute of Science and Technology, Seoul 02792, Republic of Korea
2. Cancer Research Institute, Chungnam National University, Daejeon 35015, Republic of Korea
3. Chemical & Biological integrative Research Center, Korea Institute of Science and Technology, Seoul, 02792, Republic of Korea
4. Department of Chemical and Biological Engineering, Korea University, Seoul 02841, Republic of Korea
5. Department of Biomicrosystem Technology, Korea University, Seoul 02841, Republic of Korea
6. Technological Convergence Support Center, Korea Institute of Science and Technology, Seoul, 02792, Republic of Korea
7. KU-KIST Graduate School of Conversing Science and Technology, Korea University, Seoul 02841, Republic of Korea
8. Division of Bio-Medical Science \& Technology, University of Science \& Technology, Seoul 02792, Republic of Korea
9. Department of Chemistry and Nano Science, Ewha Womans University, 52 Ewhayeodae-gil, Seodaemun-gu, Seoul 03760, Republic of Korea

† These authors contributed equally to this work.

a

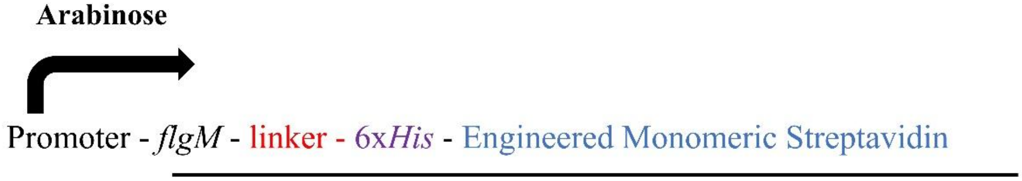

b

GGSSSSHHHHHHSGGEFASAEAGITGTWYNQHGSTFTVTAGADGNLTGQYEN
RAQGTGCQNSPYTLTGRYNGTKLEWRVEWNNSTENCHSRTEWRGQYQGGA
EARINTQWNLTYEGGSGPATEQGQDTFTKVKPSAASGSDYKDDDDK

c

GCTAGCAGGAGGTTTGACCTATGAGCATTGACCGTACCTCACCTTTGAAAC
CCGTTAGCACTGTCCAGACGCGCGAAACCAGCGACACGCCGGTACAAAAA
ACGCGTCAGGAAAAAACGTCCGCCGCGACGAGCGCCAGCGTAACGTTAAG
CGACGCGCAAGCGAAGCTCATGCAGCCAGGCGTCAGCGACATTAATATGG
AACGCGTCGAAGCATTAAAAACGGCTATCCGTAACGGTGAGTTAAAAATGG
ATACGGGAAAAATAGCAGACTCGCTCATTCGCGAGGCGCAGAGCTACTTAC
AGAGTAAAAAAGGCGGCAGCAGCACCAGCCATCATCATCATCATCATAGCG
GCGGCGAATTTGCGAGCGCGGAAGCGGGCATTACCGGCACCTGGTATAAC
CAGCATGGCAGCACCTTTACCGTGACCGCGGGCGCGGATGGCAACCTGAC
CGGCCAGTATGAAAACCGCGCGCAGGGCACCGGCTGCCAGAACAGCCCGT
ATACCCTGACCGGCCGCTATAACGGCACCAAACTGGAATGGCGCGTGGAAT
GGAACAACAGCACCGAAAACTGCCATAGCCGCACCGAATGGCGCGGCCAG
TATCAGGGCGGCGCGGAAGCGCGCATTAACACCCAGTGGAACCTGACCTAT
GAAGGCGGCAGCGGCCCGGCGACCGAACAGGGCCAGGATACCTTTACCAA
AGTGAAACCGAGCGCGGCGAGCGGCAGCGATTATAAAGATGATGATGATAA
ATAAGAGCTC

Figure S1. Schematic and sequences of flgM-streptavidin constructs. (a) Schematic diagram of the constructs, (b) flgM-streptavidin amino acid sequence, (c) flgM-streptavidin nucleotide sequence

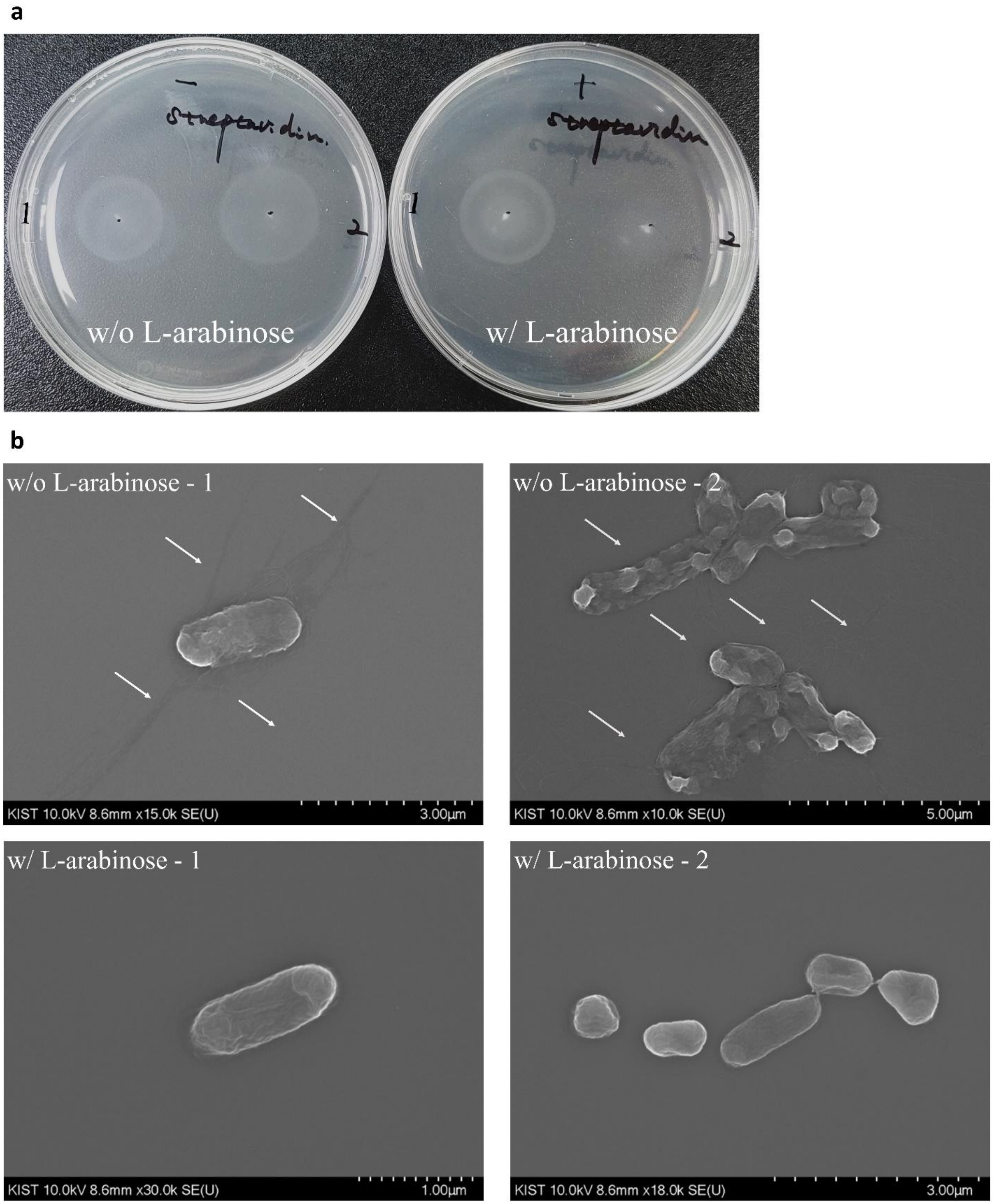

Figure S2. Streptavidin secretion validation of SAS. (a) Bacterial motility assay: (1) shows the control strain (#1237) with the pBad18 asd+ plasmid, and (2) shows the #1594 strain secreting streptavidin. The assay was performed both in the absence (left) and presence (right) of L-arabinose, with each strain cultured for 6 h at 37°C before observation. (b) Scanning electron microscope (SEM) images depicting bacterial flagella (indicated by white arrows).

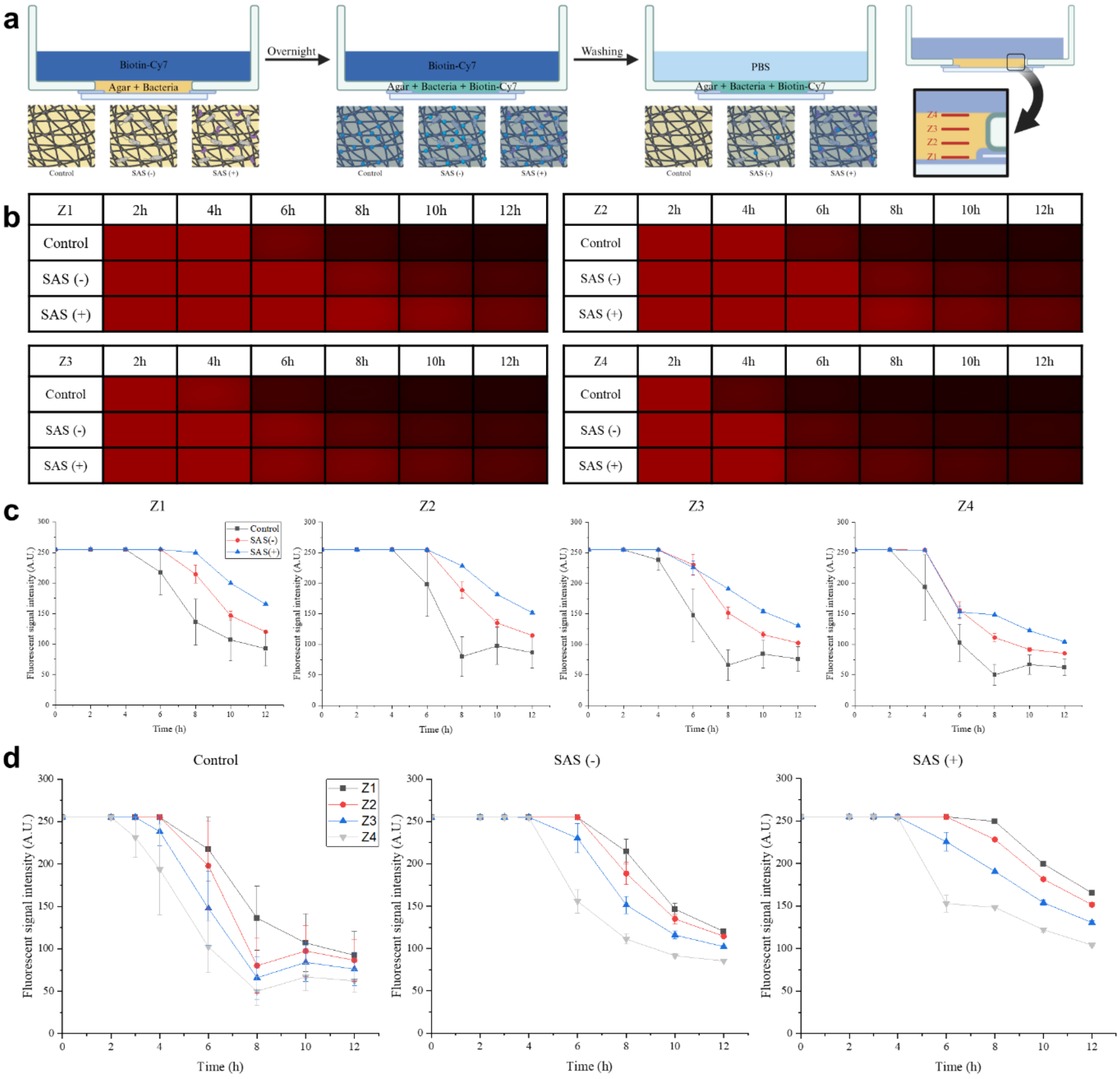

Figure S3. *In vitro* assay illustrating the interaction between bacterial-secreted streptavidin and biotin within a simulated tumor microenvironment. (a) Schematic representation of the experimental setup employing an agarose matrix. (b) Fluorescence images recorded over time at locations Z1, Z2, Z3, and Z4 within the agarose matrix depicted in (a). (c) Fluorescent signal intensity (0-255) plotted as a function of incubation time for each condition. (d) Time-dependent fluorescent signal intensity (0-255) from positions Z1 to Z4 for each location.

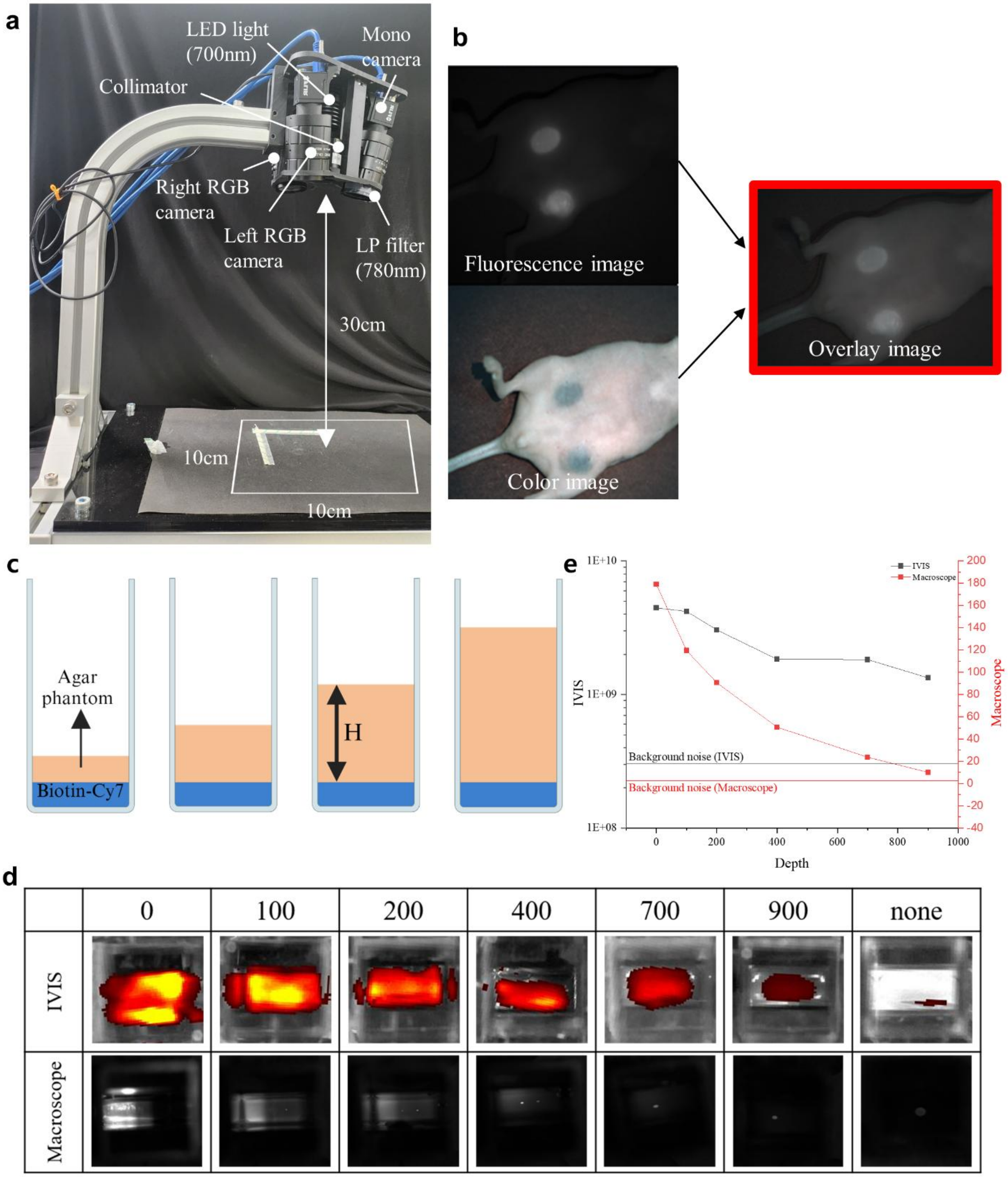

Figure S4. Analysis of fluorescent signal intensity using the macroscope. (a) Mechanical design of the macroscope, (b) Fluorescence and white light images captured by the macroscope, (c) Schematic representation of fluorescent signal intensity attenuation experiments as a function of depth, (d) Fluorescence images obtained with the IVIS system and macroscope for varying thicknesses of agar phantom (note: 'None' indicates the image with only 500 µL of agar phantom), (e) Fluorescent signal intensity relative to the depth of the agar phantom ('None' in (d) denotes background noise).

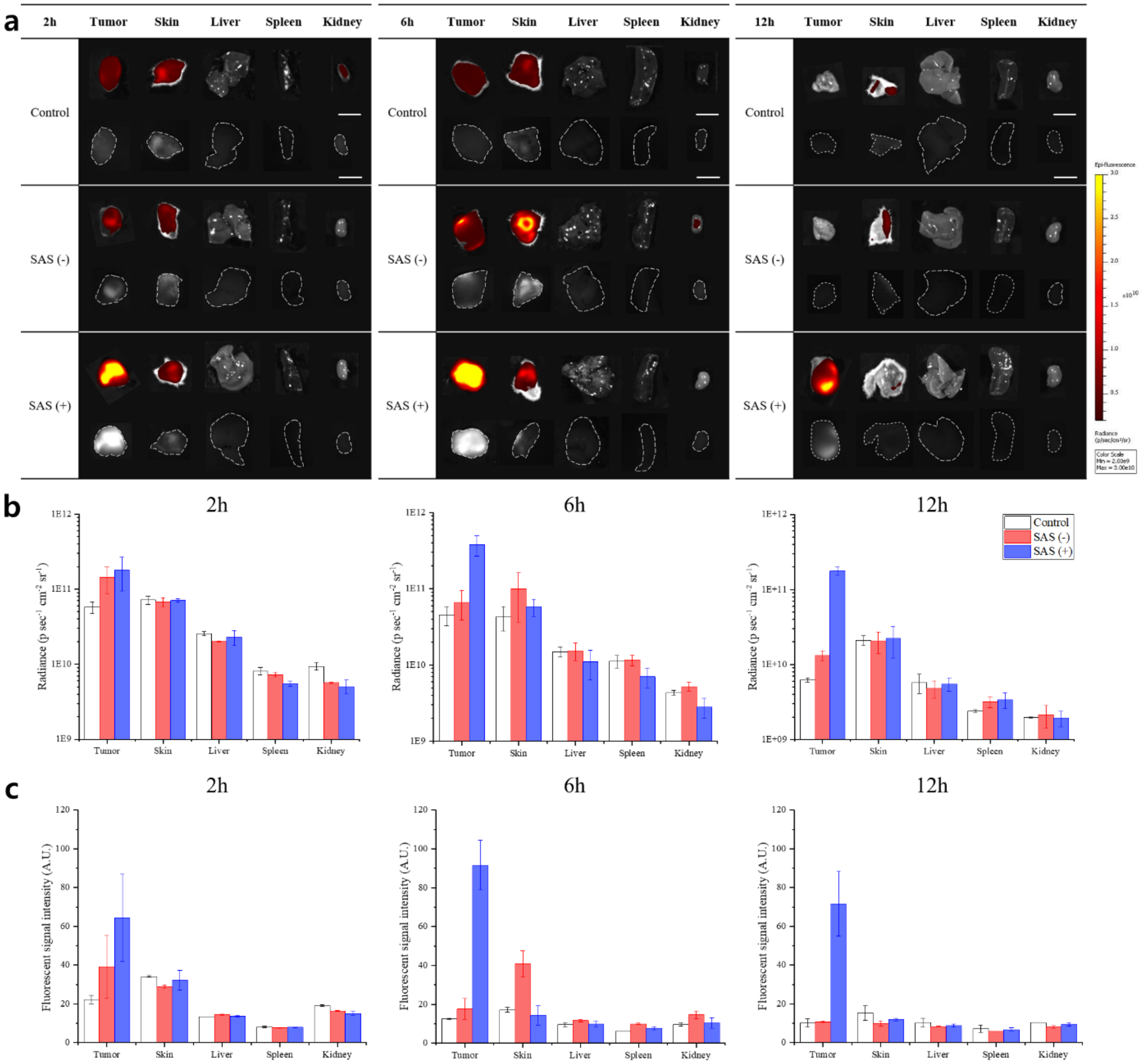

Figure S5. Intra-tumoral fluorescent signal intensity analysis. (a) Fluorescence images of tumors and organs obtained using IVIS and macroscope at 2, 6, and 12 h post-incubation (Scale bar = 1 cm). (b) Mean fluorescent signal intensity recorded by IVIS (n=3). (c) Mean fluorescent signal intensity (0-255) recorded by macroscope (n=3).

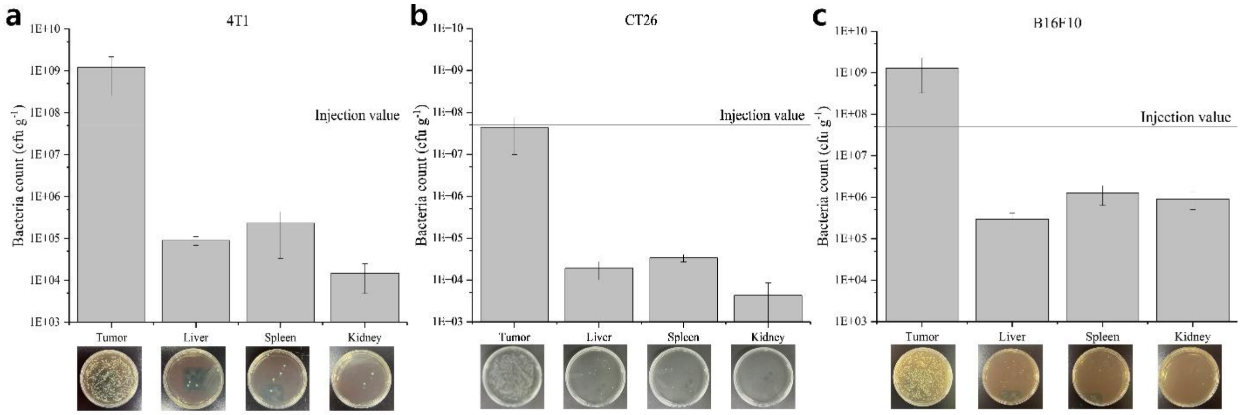

Figure S6. Bacterial cfu in tumor and organs. The agar plates used for tumor samples were diluted at 10^{-3}, while those for organ samples were diluted at 10^{-1}. Data are presented for three cancer cell lines: (a) 4T1, (b) CT26, and (c) B16F10 (n=3).

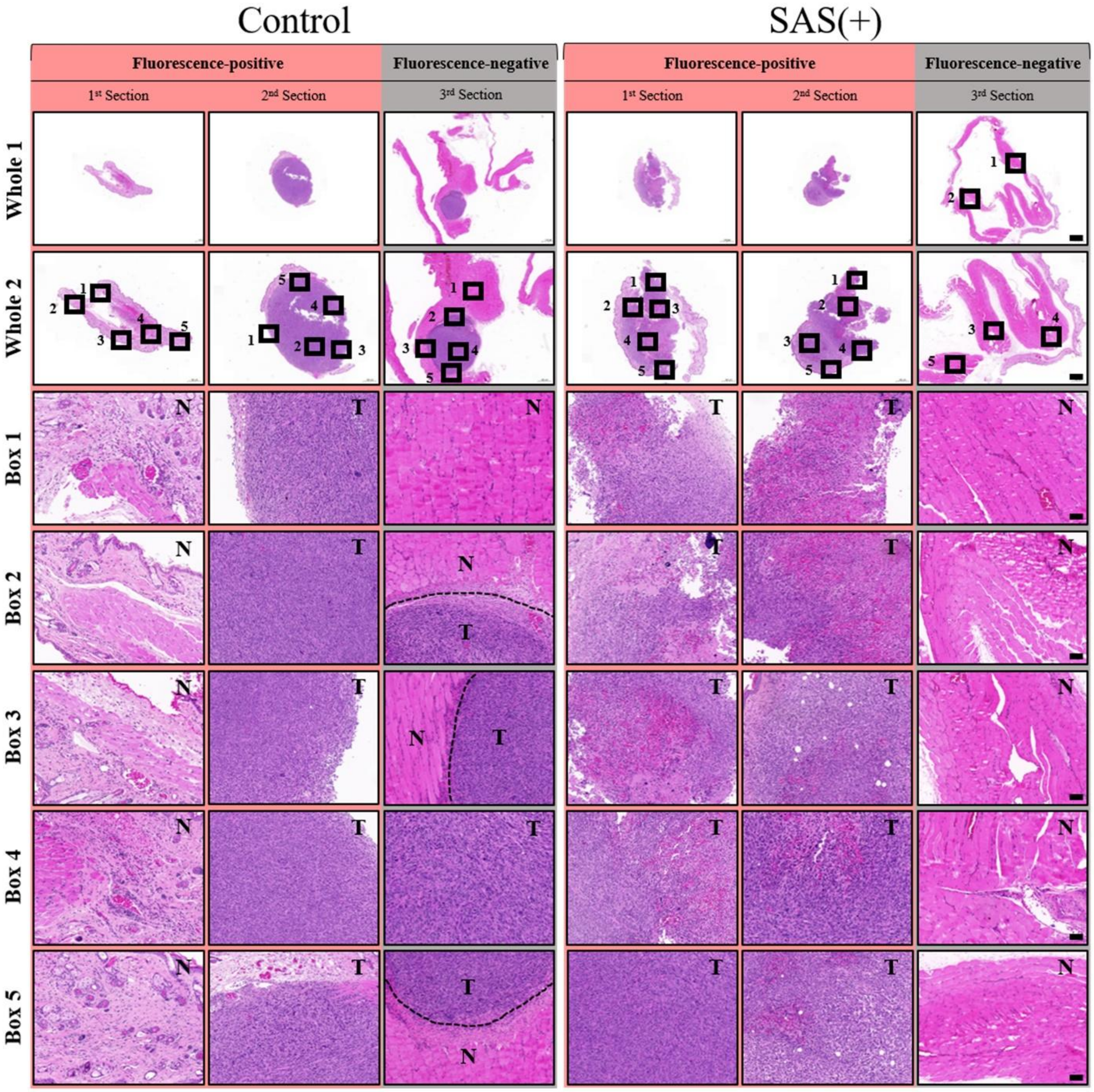

Figure S7. H&E staining results showing magnified views of the sections indicated by the boxes (T: Tumor, N: Normal tissue). (Whole 1 scale bar, 1000um. Whole 2 scale bar, 500um. Box scale bar, 50um.)

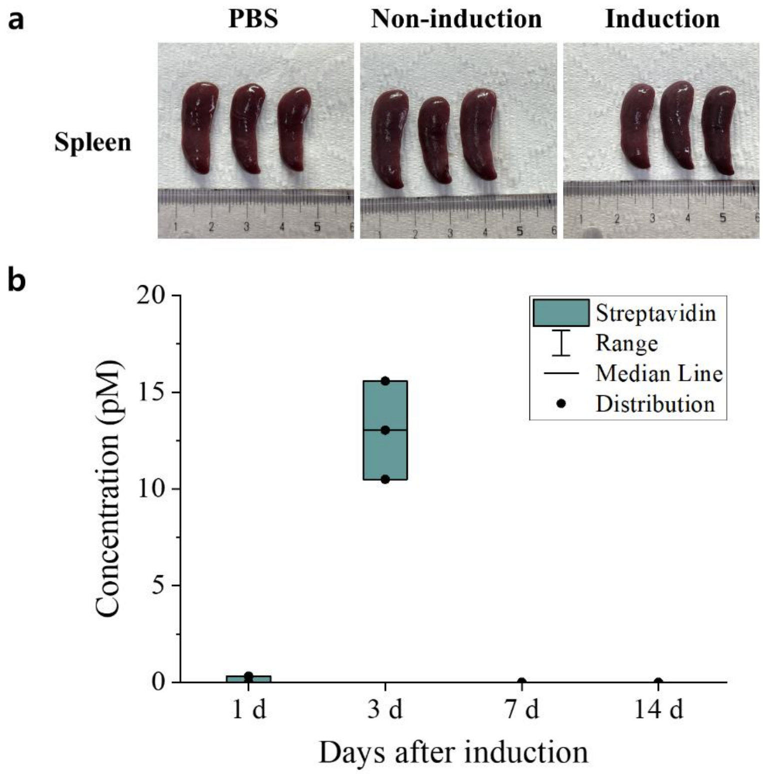

Figure S8. (a) Images of spleens from tumor-bearing mice after *Salmonella* treatment. Spleens from each group were collected on day 15. (b) Streptavidin concentration in blood and urine from tumor-bearing mice after induction (n=3).

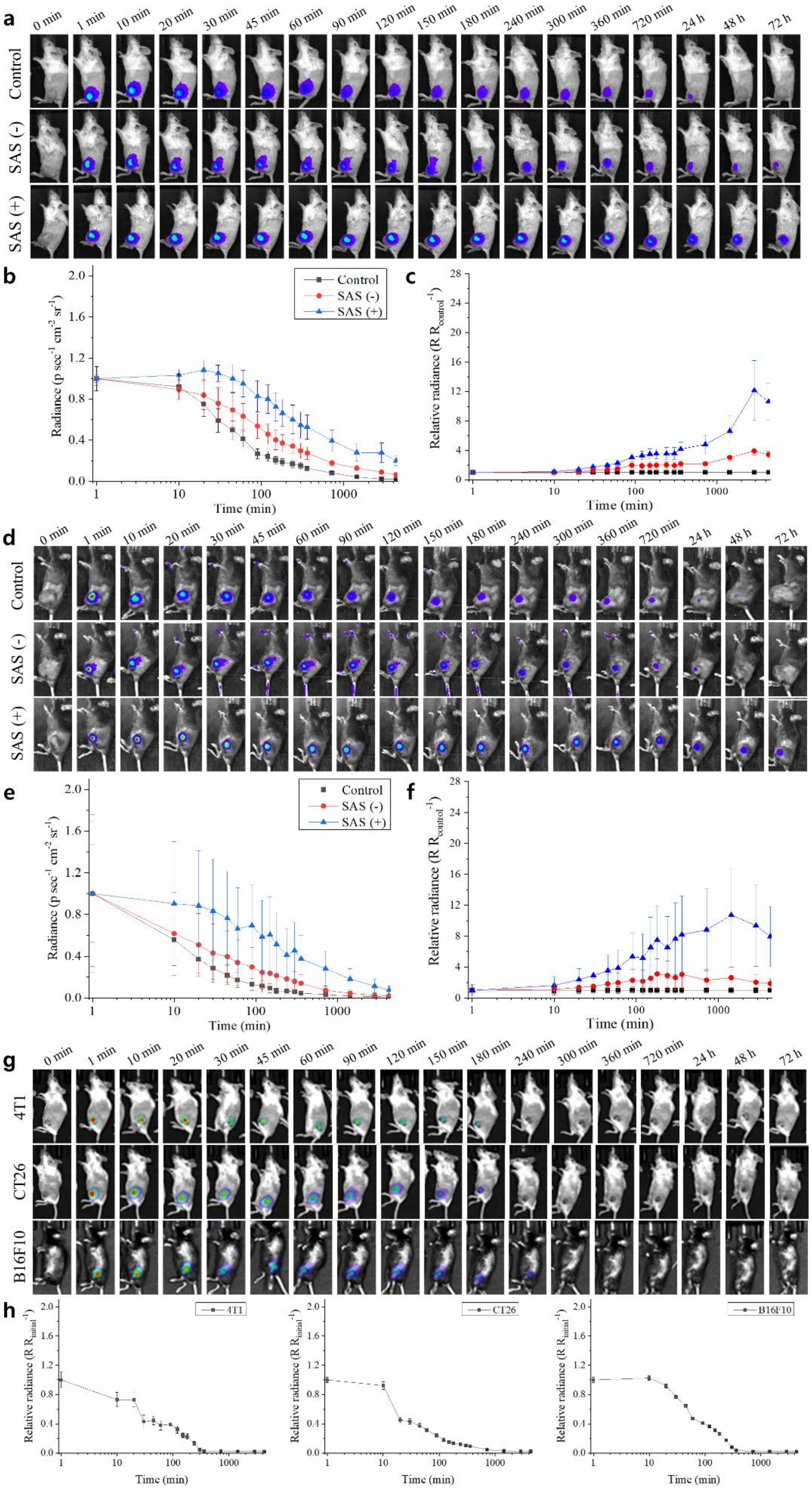
a
0 min
1 min
10 min
20 min
30 min
45 min
60 min
90 min
120 min
150 min
180 min
240 min
300 min
360 min
720 min
24 h
48 h
72 h
Control
SAS (-)
SAS (+)
b
Radiance (p sec^{-1} cm^{-2} sr^{-1})
Time (min)
Control
SAS (-)
SAS (+)
c
Relative radiance (R $R_{control}^{-1}$)
Time (min)
d
Control
SAS (-)
SAS (+)
e
Radiance (p sec^{-1} cm^{-2} sr^{-1})
Time (min)
Control
SAS (-)
SAS (+)
f
Relative radiance (R $R_{control}^{-1}$)
Time (min)
g
4T1
CT26
B16F10
h
Relative radiance (R $R_{initial}^{-1}$)
Time (min)
4T1
CT26
B16F10

Figure S9. Time-dependent fluorescent signal intensity and IVIS Imaging in CT26- and B16F10-bearing mice. (a) IVIS images of CT26-bearing mice over time (filter set: λ_{exc} = 745 nm, λ_{emi} = 800 nm) (n=3). (b) Time-dependent fluorescent signal intensity in the tumor from (a), normalized to the initial radiance value. (c) Relative fluorescent signal from (a) normalized to the control group. (d) IVIS images of B16F10-bearing mice over time (same filter set) (n=3). (e) Time-dependent fluorescent signal intensity in the tumor from (d), normalized to the initial radiance value. (f) Relative fluorescent signal from (d), normalized to the control group. (g) IVIS images of 4T1-, CT26- and B16F10-bearing mice over time (filter set: λexc = 735 nm, λemi = 830 nm) (n=3). (h) Relative fluorescent signal from (g), normalized to the initial value.

Movie S1. Intraoperative demonstration of tumor resection in 4T1 orthotopic tumor mice using a Fluorescent Macroscope. The video compares tumor visualization and removal between the Control group with conventional contrast agents and the SAS(+) group with bacterial-based fluorescent contrast agents.

Table S1. *Salmonella* strains

Number	Name	Relative genotype & information	Used
**#26**	*Salmonella enterica* subsp. enterica serovar *Typhimurium* str. LT2	Taxonomy ID: 99287(ATCC 700720)	Lab strain
**#46**	SF586	Transformation host when transferring from E. coli to *Salmonella*	From Professor In soo Lee at Hannam University
**#178**	*S. typhimurium* BRD 509 (BRD 509)	mutant aroA/aroD variant of *Salmonella* strain SL1344	From Professor In soo Lee at Hannam University
**#177**	BRD 509 asd *salmonella*	mutant aroA/aroD/asd variant of *Salmonella* strain SL1344	From Professor In soo Lee at Hannam University
**#245**	BRD 509 rcsB *salmonella*	mutant aroA/aroD/rcsB variant of *Salmonella* strain SL1344	In this study
**#1237**	BRD 509 asd rcsB *salmonella*	mutant aroA/aroD/asd/rcsB variant of *Salmonella* strain SL1344	In this study
**#1594**	flgM-streptavidin-flhDC-pBAD18-asd+ / #1237	flgM-streptavidin-flhDC-pBAD18-asd+ / #1237 (aroA aroD asd rcsB-)	In this study

Table S2. Time-dependent release profile curve fitting parameters

		**Control**				**SAS (-)**				**SAS (+)**			
		**A_1**	**A_2**	**A_3**	**A_4**	**A_1**	**A_2**	**A_3**	**A_4**	**A_1**	**A_2**	**A_3**	**A_4**
**4T1**	A	0.6020	0.2689	0.0928	0.0364	0.3349	0.4202	0.2449	-	0.2162	0.2555	0.5284	-
	T	8.53	28.38	185.04	1171.70	8.56	66.88	875.64	-	143.91	541.66	16727.45	-
**CT26**	A	0.7805	0.1267	0.0928	-	0.4521	0.3732	0.1747	-	0.6031	0.3969	-	-
	T	39.43	350.0	994.9	-	50.6	284.4	4100.3	-	266.5	11499.2	-	-
**B16F10**	A	0.2711	0.4573	0.2153	0.0563	0.3921	0.2826	0.2546	0.0705	0.0747	0.2643	0.2165	0.4446
	T	4.71	14.50	71.64	712.5	4.54	29.65	245.3	1544.9	17.61	124.1	689.2	24831.1

Table S3. Time-dependent release profile curve fitting parameters of the ICG

		**Control**			
		**A_1**	**A_2**	**A_3**	**A_4**
**4T1**	A	0.3275	0.6725	-	-
	T	7.29	124.4	-	-
**CT26**	A	0.4625	0.3509	0.1866	-
	T	14.55	46.60	306.79	-
**B16F10**	A	1	-	-	-
	T	98.96	-	-	-

Table S4. Primers used in this study

Primer name	Sequence
**rcsB-pkd13-For**	ATCAGCGACATTGACGCCTACGTCAAAAGCTTGCTGTAGCGTGTAGGCTGGAGCTGCTTCGAAGTT
**rcsB-pkd13-Re**	CCTGATAAGCGTAGCGCCATCAGGCTGGGTAACATAAAAGCTGTCAAACATGAGAATTAATTCCGGGGA
**flhD-Sac1-for**	TCGATCGAGCTCAGGAGGTTTGATCCTATGGGAACAATGCATACATCCGAGTTGCT
**flhC-Sal1-re**	TCGATCGTCGACTTAAACAGCCTGTTCGATCTGTTCATCCAGCAGTT
**asd-ClaI-For**	GGTGCATCGATCAGGAAAAAAACGCTATGAAAAATGTTGGT
**asd-ClaI-RE**	TCGATCATCGATCTACGCCAACTGGCGCAGCATTCGACGCAGCG
